# Case-control study of risk factors for abscess development following hepatitis B vaccination in children in Timor-Leste, 2024

**DOI:** 10.1371/journal.pone.0351879

**Published:** 2026-06-22

**Authors:** Mariano da Silva Marques, Filipe de Neri Machado, Marcelo Amaral Mali, Nevio da Costa Sarmento, Domingos Soares, Noel Gama Soares, Cristovão de Alexandria Barros, Livia Natalia Maria Guterres Babo, Armandinha A. da C. Silva, Evaliza Adão Mendonça, Natalia Jesus da Silva, Nazario Barreto dos Santos, Marck A. A. Magno Neves

**Affiliations:** 1 Instituto Nacional da Saúde Pública, Dili, Timor-Leste; 2 Universitas Sebelas Maret, Surakarta, Jawa Tengah, Indonesia; Federal University Otuoke, NIGERIA

## Abstract

**Background:**

Hepatitis B vaccination is essential for preventing chronic hepatitis B infection and its long-term complications. In 2024, a high proportion of reported adverse events following immunization (AEFI) in Timor-Leste involved injection-site abscesses among infants who received the hepatitis B birth dose. This study aimed to explore potential factors associated with abscess formation to inform improvements in immunization practices.

**Methods:**

We conducted a retrospective matched case-control study in public health facilities across 10 municipalities in Timor-Leste. Cases were infants aged <12 months with a clinically confirmed injection-site abscess occurring within 14 days after receiving the hepatitis B birth dose in 2024. Each case was matched 1:1 with a control by vaccination facility, approximate vaccination date, infant age group, and sex. Data on recipient characteristics, provider practices, vaccine handling, and cold chain management were collected through caregiver interviews, review of clinical and vaccination records, and facility assessments. Matched bivariate associations were evaluated using the exact McNemar test.

**Results:**

None of the examined variables showed statistically significant associations with injection-site abscess formation; confidence intervals were extremely wide, reflecting the small sample size (12 pairs) and the very small number of discordant pairs (≤4 for all comparisons). Findings should therefore be interpreted as descriptive and hypothesis-generating only.

**Conclusions:**

This exploratory matched case-control study did not identify any statistically significant risk factors for injection-site abscesses following hepatitis B birth-dose vaccination. Due to the very small sample size, inferential estimates were highly unstable; therefore, the findings should be interpreted as descriptive and hypothesis-generating rather than confirmatory. Despite these limitations, strengthening safe injection practices, appropriate multidose vial management, and adverse events following immunization (AEFI) surveillance systems remains critical. Larger prospective studies are needed to reliably investigate potential determinants of abscess formation.

## Introduction

Hepatitis B is a major global health problem. The virus can cause immediate, severe illness and, for millions, leads to a lifelong infection that slowly damages the liver. This ongoing burden of disease takes a heavy toll worldwide, resulting in a significant number of premature deaths annually. In 2019, the World Health Organization (WHO) estimated that 296 million people were living with chronic hepatitis B infection, which caused over 820,000 deaths [1,2]. The cornerstone for altering this epidemiological trajectory is the availability of safe and highly effective prophylactic vaccines against the virus. These vaccines confer protection against initial hepatitis B virus (HBV) infection and are critically effective in preventing the subsequent development of the chronic carrier state, which is the primary driver of severe long-term sequelae and mortality [3]. Since its initial approval in 1982, the global administration of the hepatitis B vaccine has exceeded one billion doses [2]. Parental vaccine hesitancy and refusal are often driven by mistrust of vaccine safety profiles and specific concerns regarding potential adverse reactions [4].

Adverse Events Following Immunization (AEFI) are defined by the WHO as any untoward medical occurrence that follows immunization and does not necessarily have a causal relationship with the use of the vaccine. Adverse events may be any unfavorable or unintended signs, abnormal laboratory findings, symptoms, or diseases [5]. AEFIs can be classified into five categories: vaccine product–related reactions, vaccine quality defect–related reactions, immunization error–related reactions, immunization anxiety–associated reactions, and coincidental events [6]. Globally, the number of reported AEFI differs by country. According to WHO estimates, the incidence of serious AEFIs is approximately 1 per 100,000 doses administered, whereas non-serious AEFIs are more common and often underreported [7]. Injection-site reactions, such as swelling, redness, and abscesses, are among the most frequently reported non-serious events, particularly in low-resource settings where cold chain and aseptic techniques may be compromised.

The hepatitis B vaccine is widely recognized for its safety [8], being associated primarily with mild and self-limited adverse events [9]. The most common adverse reactions to the hepatitis B vaccine are local and transient systemic symptoms, typically occurring within 24 hours of administration. These include pain at the injection site (with an incidence of 3–29 per 100 doses), erythema (3 per 100), and swelling (3 per 100). Mild systemic symptoms such as fever (1–6 per 100) and headache (3 per 100) have also been documented [9–11]. Severe reactions such as anaphylaxis occur at an estimated rate of approximately 1–2 cases per million vaccine doses administered [9,12]. Notably, declines in hepatitis B vaccination coverage have been observed following media reports of infant deaths after administration of the hepatitis B vaccine birth dose (HepB-BD) in Vietnam [13] and China [14,15]. As post-marketing data accumulate, several serious adverse events following hepatitis B vaccination have been reported. The most recent pharmacovigilance analysis in the United States suggests that adverse events associated with the hepatitis B vaccine include general disorders and administration site conditions, nervous system disorders, aplastic anaemia, exfoliative dermatitis, and haemolytic anaemia [16]. However, quantitative studies based on real-world data remain limited, and comprehensive information on these adverse events is still lacking [16].

In the Southeast Asia Region (SEARO), AEFIs have been increasingly documented, particularly in the context of expanding immunization coverage. A 2020 regional review found that injection-site abscesses were frequently reported AEFIs for DTP and hepatitis B vaccines given in the EPI program. In India, for example, multiple clusters of injection-site abscesses have been linked to programmatic errors, including improper needle reuse, multidose vial contamination, and breaches in cold chain maintenance [17].

In Timor-Leste, public healthcare, including routine immunization of children, is provided free of charge to nationals. The three-dose infant hepatitis B vaccination series was introduced in Timor-Leste in 2007, followed by the hepatitis B birth dose in 2016 [18]. According to the WHO/UNICEF joint estimates of national immunization coverage (WUENIC), hepatitis B at birth-dose coverage is 76% from the year 2021–2024 [19]. National AEFI surveillance remains limited in Timor-Leste, with significant underreporting and a lack of publicly available systematic data on the incidence and nature of the adverse events. However, anecdotal reports and clinical observations suggest a concerning trend of abscess formation following hepatitis B vaccine administration, particularly when delivered from multidose vials.

According to the national surveillance data for 2024, 62 cases of AEFI were reported. Within this cohort, 89% of cases involved abscess formation, with the affected demographic predominantly comprising children under the age of one year [20]. These events occurred during routine immunization. Of the total reported AEFI cases, 95% were classified as serious [20]. Furthermore, the surveillance data documented two fatal cases. These events pose serious threats to community trust in vaccination programs and highlight critical evidence gaps in the literature. To date, no published national study has systematically investigated the risk factors associated with these abscesses in children.

Multidose vials present considerable advantages, including lower packaging and storage costs and reduced medical waste. However, this efficiency must be carefully weighed against the persistent concern of safeguarding against contamination caused by human error during administration [21]. Therefore, investigating the determinants of abscess formation is therefore essential to inform evidence-based interventions, strengthen immunization safety, and restore public confidence.

This study aimed to explore potential factors associated with abscess occurrence during an AEFI investigation following hepatitis B vaccination using multidose vials in children in Timor-Leste.

## Methods

### Study design and Setting

We conducted a retrospective matched case–control study to investigate factors associated with injection-site abscess following hepatitis B birth-dose vaccination in Timor-Leste in 2024. The study was conducted in public health facilities across 10 municipalities where adverse events following immunization (AEFI) had been reported through the national surveillance system.

This investigation was undertaken as part of the national AEFI response to identify potential programmatic or clinical factors associated with abscess formation following routine hepatitis B vaccination.

The choice of risk factors to investigate was based on a review of the common breaches in safe injection practices and vaccine management. We categorized these factors as provider practices (including skin disinfection and training on proper vaccine administration [22]); Cold Chain Maintenance, including temperature monitoring and vaccine storage conditions [23,24]); Vaccine Handling, including repeated vial puncture [25,26] Vaccinator Factors (including training [27] and work experience [28] and recipient factors, including age [28], weight [29,30], and gestational age [31].

### Study cases

Cases were infants under 12 months who developed a clinically diagnosed injection-site abscess—characterized by local swelling and signs of inflammation (with or without drainage)—within 14 days post-vaccination, as documented by a healthcare professional. Diagnosis followed clinical examination and national AEFI forms; case identification relied on the Ministry of Health’s AEFI surveillance database.

In 2024, a total of 62 adverse events following immunization (AEFI) were reported through the national surveillance system in Timor‑Leste. All reported AEFI cases were initially screened for eligibility. Cases were first restricted to infants younger than 12 months who had received the hepatitis B birth‑dose vaccine during routine immunization services and were reported to have developed an injection‑site abscess within 14 days of vaccination. Records were then reviewed to verify the clinical diagnosis of abscess and to confirm the availability of vaccination documentation. Cases were further excluded if vaccination records were incomplete, the diagnosis could not be verified, or caregivers could not be contacted for interview. Following application of these eligibility criteria and the availability of suitable matched controls from the same vaccination facilities and time periods, 12 cases with complete data were retained and successfully matched 1:1 with 12 controls for the final analysis

**Fig 1:**
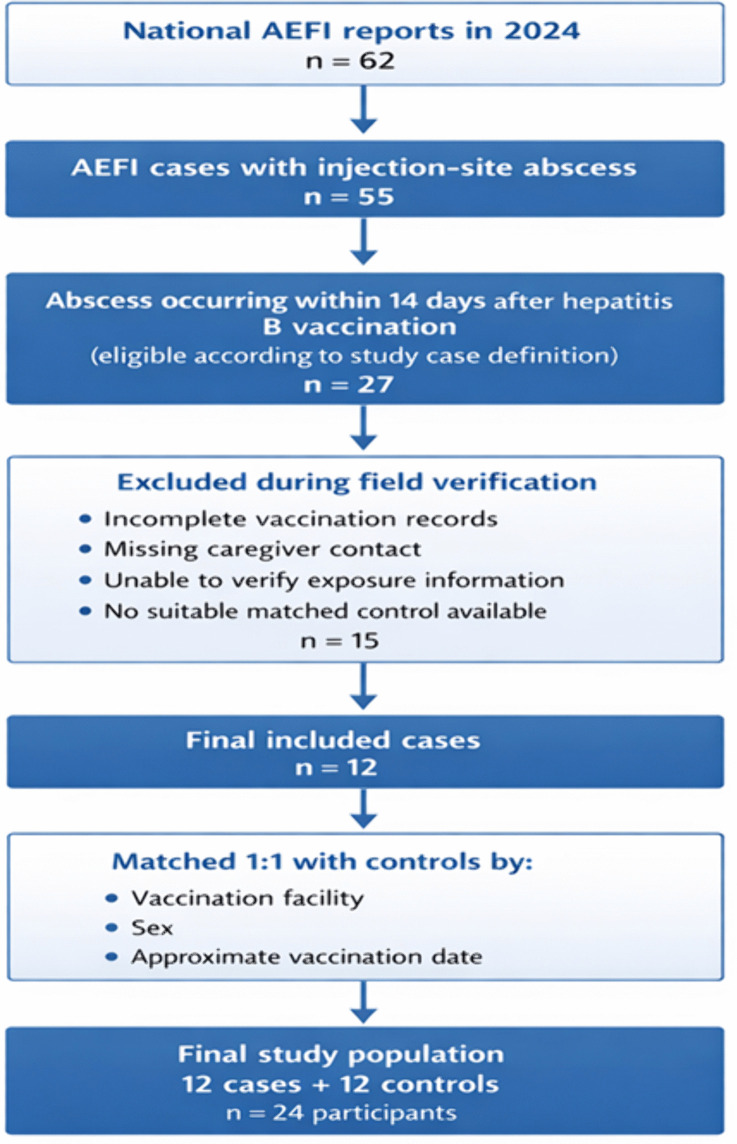
Case selection flow diagram. A total of 62 adverse events following immunization (AEFI) were reported through the national surveillance system in Timor-Leste in 2024. After restricting to infants aged <12 months with a clinically confirmed injection-site abscess occurring within 14 days after hepatitis B birth-dose vaccination and verifying availability of vaccination records and caregiver contact, 12 cases met eligibility criteria. Each case was matched 1:1 with a control by vaccination facility, approximate vaccination date (±7 days), infant age group, and sex, resulting in 12 matched pairs for final analysis.

Selection of study participants from the national adverse events following immunization (AEFI) surveillance database in Timor-Leste, 2024. Cases were restricted to infants with clinically confirmed injection-site abscess occurring within 14 days after hepatitis B birth-dose vaccination. After applying eligibility criteria and matching requirements, 12 cases and 12 matched controls were included in the final analysis. The flow diagram (Fig 1) has been added to visually represent this process.

### Study controls

Controls were infants who received the hepatitis B birth-dose vaccine during the same time period and at the same health facility as the corresponding case but did not develop any reported adverse event following immunization during the observation period.

For each eligible case, one control was selected from the same health facility vaccination register. Controls were infants who received the hepatitis B birth-dose vaccine during the same period but did not develop any reported AEFI during the 14-day observation period.

Controls were matched to cases based on vaccination facility, approximate vaccination date (±7 days), infant age group, and sex to control for facility‑level practices and temporal variation in immunization services.

This matching strategy was intended to control for facility-level practices, vaccine storage conditions, and temporal variation in immunization services.

### Data collection

The data collection period for this retrospective study was from November 4 to December 14, 2025.

Data were collected from multiple sources, including vaccination records and clinical documentation, structured interviews with caregivers, interviews with healthcare workers involved in vaccine administration, and facility‑level assessments of vaccine storage and immunization practices.

Information collected included infant demographic characteristics, birth history, vaccination procedures, vaccine storage practices, and healthcare worker experience.

### Exposure variables and operational definitions

Exposure variables related to vaccination practices, vaccine handling, cold chain maintenance, and recipient characteristics were evaluated using predefined binary operational definitions (Table 1). Provider practices included skin disinfection and vaccinator training status. Vaccine handling variables captured the use of newly opened versus previously opened multidose vials. Cold chain maintenance was assessed through retrospective review of temperature monitoring logs and storage conditions in accordance with national guidelines applicable in 2024. Recipient‑related factors included sex, birth weight, and gestational age at delivery.

**Table 1:** Operational definitions of exposure variables.

Variable	Operational Definition	Measurement
**Provider Practice**		
Skin Disinfection	The application of an antiseptic at the vaccination site immediately before administration, strictly according to the standard protocol.	Yes / No
**Vaccine Handling**		
Multidose Vial Policy	Whether the administered vaccine dose was taken from a vial opened for the first time at the vaccination session or from a vial that had been previously opened and stored according to the WHO multidose vial policy	Newly opened / Previously opened
**Cold Chain Maintenance**		
Temperature Monitoring	Documentation of vaccine storage temperature monitoring at least twice daily, recorded in facility temperature logs according to national cold chain guidelines.	Proper / Improper
Vaccine Storage Conditions	Vaccine storage conditions evaluated according to national EPI guidelines, including maintenance of recommended temperature range (2–8°C), absence of freezing, protection from direct light, and appropriate refrigerator storage practices.	Appropriate / Inappropriate
**Recipient and Vaccinator Factors**		
Sex	The biological sex of the vaccine recipient.	Male / Female
Weight	The weight of the recipient at the time of vaccination.	<2500 grams />2500 grams
Gestational Age [31]	The completed weeks of gestation at the time of delivery.	<37 weeks / ≥ 37 weeks
Vaccinator Status	The employment status of the healthcare worker who administered the vaccine.	Permanent staff / Non-permanent staff
Vaccinator Experience	The number of years of professional experience the vaccinator has in providing immunization services.	<2 years / ≥ 2 years
Vaccinator Training	Whether the vaccinator has received formal training on proper vaccine administration procedures.	Yes / No
Injection Site	The specific anatomical site on the recipient’s body where the vaccine was injected.	Left Thigh / Right Thigh
**Outcome**		
Abscess Formation	A clinically confirmed presence of an abscess (a collection of pus) at the injection site, as diagnosed by a healthcare professional within 14 days following vaccination.	Yes / No

### Statistical analysis

Statistical analysis was performed using SPSS version 29.0 (IBM Corp., Armonk, NY, USA). Because the study used 1:1 matching, associations between potential risk factors and abscess occurrence were assessed using the exact McNemar test. Matched odds ratios (mORs) with 95% confidence intervals (CIs) were calculated based on discordant pairs. Variables with no variation across matched pairs were excluded from the matched analysis because no discordant pairs were available for comparison. Given the very small number of discordant pairs, inferential estimates derived from the matched analysis were considered statistically unstable and are presented for descriptive completeness only.

### Ethics and informed consent

This retrospective study was granted ethical approval by the Institute National of Public Health Timor-Leste — Research Ethics and Technical Committee (INSP TL-RETC) in 2025 (Ref. No.: 1952/INSP-TL/UEPD-AL/IX/025).

Informed consent was obtained in writing from a parent or legal guardian of all participating children. This consent process permitted the interview with the caregiver and the retrospective examination of the child’s health records, vaccination logs, and AEFI forms from 2024. Participation was voluntary, and caregivers were informed of their right to refuse or withdraw without any repercussions for their child’s care.

Data obtained were managed in a secure, password-protected Microsoft Excel database.

## Results

### Sociodemographic Characteristics

There were 24 participants (12 cases and 12 matched controls) (Table 2). The sex distribution was identical between groups, with males comprising 58.3% of both cases and controls. A higher proportion of controls had low birth weight (<2500 g) compared to cases (16.7% vs. 8.3%). Similarly, more controls were born preterm (<37 weeks) than cases (25.0% vs. 16.7%). However, because this study used a matched design, crude group frequencies do not determine the estimated association. Matched analysis is based solely on discordant pairs (pairs in which the case and control differ in exposure status). Therefore, the direction and magnitude of the odds ratio reflect within-pair differences rather than overall exposure prevalence in each group. Permanent staff members administered most vaccinations in both groups (cases: 75.0%, controls: 83.3%). The left thigh was the most common injection site for both groups.

**Table 2:** Sociodemographic characteristics of cases and controls.

Variable	Case (n = 12) n (%)	Control (n = 12) n (%)
**Sex**		
Female	5 (41.7)	5 (41.7)
Male	7 (58.3)	7 (58.3)
**Birth weight (g)**		
<2500	1 (8.3)	2 (16.7)
≥2500	11 (91.7)	10 (83.3)
**Gestational age (weeks)**		
<37	2 (16.7)	3 (25.0)
≥37	10 (83.3)	9 (75.0)
**Vaccinator status**		
Permanent staff	9 (75.0)	10 (83.3)
Non-permanent staff	3 (25.0)	2 (16.7)
**Injection site**		
Left thigh	7 (58.3)	8 (66.7)
Right thigh	5 (41.7)	4 (33.3)

### Provider practice

Provider practices are summarised in (Table 3) Application of antiseptic according to protocol was reported in 91.7% of both cases and controls. A lower proportion of cases received their vaccine dose from a newly opened multi-dose vial compared to controls (58.3% vs. 83.3%). The distribution of vaccinator work experience was identical between groups, with 66.7% in each group vaccinated by staff with at least two years of experience. A slightly higher proportion of cases than controls were vaccinated by staff with formal training (58.3% vs. 50.0%).

**Table 3:** Provider practice during vaccination.

Variable	Case (n = 12) n (%)	Control (n = 12) n (%)
**Antiseptic applied at vaccination site according to protocol**		
Yes	11 (91.7)	11 (91.7)
No	1 (8.3)	1 (8.3)
**Vaccinator work experience (years)**		
<2	4 (33.3)	4 (33.3)
≥2	8 (66.7)	8 (66.7)
**Vaccinator trained on proper vaccine administration**		
Yes	7 (58.3)	6 (50.0)
No	5 (41.7)	6 (50.0)
**Dose from newly opened multidose vial**		
Yes	7 (58.3)	10 (83.3)
No (previously opened)	5 (41.7)	2 (16.7)

### Vaccine storage and handling

Vaccine storage and handling practices were uniformly documented as compliant across all participating facilities. For both cases and controls, temperature monitoring logs indicated regular documentation, and physical storage conditions met national cold chain requirements. As no variability was observed in these indicators, they were excluded from the matched analysis.

### McNemar analysis for matched pairs

In the matched bivariate analysis using the exact McNemar test (Table 4), none of the examined variables showed statistically significant associations with injection-site abscess formation. However, the interpretation of matched odds ratios, confidence intervals, and p-values is severely limited by the very small number of discordant pairs observed for all variables.

**Table 4:** Matched bivariate analysis using the exact McNemar test.

Variable	Comparison	Discordant Pairs (Case + /Control-)	Discordant Pairs (Case-/Control+)	Matched Odds Ratio (mOR)	95% CI	p-value
**Birth weight**	≥2500 g vs < 2500 g	2	1	2.00	0.18–22.06	1.000
**Gestational age**	<37 weeks vs ≥ 37 weeks	1	2	0.50	0.05–5.51	1.000
**Injection site**	Left vs Right thigh	3	2	1.50	0.25–8.98	1.000
**Antiseptic application**	Yes vs No	1	1	1.00	0.06–15.99	1.000
**Vaccinator training**	Trained vs Untrained	3	2	1.50	0.25–8.98	1.000
**Vaccinator experience**	≥2 years vs < 2 years	2	2	1.00	0.14–7.10	1.000
**Vaccinator employment**	Permanent vs Non-permanent	2	1	2.00	0.18–22.06	1.000
**Multidose vial status**	Previously opened vs Newly opened	4	1	4.00	0.45–35.79	0.375

*Due to the very small number of discordant pairs (≤4 for all variables), matched odds ratios, confidence intervals, and p-values are statistically unstable and should not be interpreted as reliable measures of effect magnitude or evidence for or against association.

† Cold chain variables (temperature monitoring and physical storage conditions) were excluded from matched analysis because no discordant pairs were observed across matched case–control pairs.

For multidose vial status, the discordant pair pattern showed that in four matched pairs, the case received the vaccine from a previously opened vial, whereas the control received the vaccine from a newly opened vial; in comparison, one pair showed the opposite pattern. This distribution is compatible with a wide range of possible effect sizes and cannot reliably establish or exclude an association.

For all remaining variables, discordant pair counts ranged from 1 to 3 in either direction, indicating minimal information available for estimating associations. Variables related to cold chain maintenance (temperature monitoring and physical storage conditions) showed no variation across matched pairs and were therefore excluded from the matched analysis.

## Discussion

This matched case–control study explored potential risk factors associated with injection-site abscess following hepatitis B birth-dose vaccination in Timor-Leste. Given the small sample size, this study is inherently descriptive and hypothesis-generating; no causal inference can be drawn. In the primary matched bivariate analysis using the exact McNemar test, none of the examined variables—including infant characteristics (birth weight, gestational age), provider practices (antiseptic use, vaccinator training), or vaccine handling (multidose vial status)—were significantly associated with abscess occurrence. The discordant pair pattern for multidose vial status (4 vs. 1) is compatible with a very wide range of possible effects, from no association to a substantial increase in risk. No meaningful inference can be drawn from this pattern. The wide confidence intervals observed for all associations reflect substantial statistical uncertainty, which is expected given the small number of matched pairs (n = 12) and the limited number of discordant observations.

The absence of statistically significant associations should not be interpreted as evidence of no risk. The small number of matched pairs limited statistical power and produced unstable estimates, reducing the ability to detect or exclude meaningful associations. Potential outcome misclassification and recall bias in this retrospective study may also have biased associations toward the null. Accordingly, these findings should be interpreted as exploratory and hypothesis-generating, with primary value in informing future studies and reinforcing safe vaccination practices.

The discordant pair distribution for multidose vial status (4 pairs in which the case received vaccine from a previously opened vial and 1 pair with the opposite pattern) does not permit a reliable estimation of association because of the very small sample size and resulting statistical instability. Although contamination risks associated with multidose vial handling are biologically plausible and have been discussed in previous literature [21,25]. No meaningful inference can be drawn from this pattern.

Birth weight and gestational age also showed no significant associations. Although low birth weight and prematurity have been hypothesized to influence tissue characteristics or immune responses [29–31], this study was underpowered to detect such effects. The small number of preterm or low-birth-weight infants in the sample, and the even smaller number of discordant pairs, precludes any meaningful inference about these biological factors.

Other vaccination-related factors—including injection site, vaccinator training, and experience—were not associated with abscess formation. The lack of association may indicate that formal documentation of training or years of service does not necessarily correlate with consistent adherence to aseptic techniques in real-world settings [27]. Similarly, the absence of variation in documented cold chain practices may reflect satisfactory compliance, but it could also indicate that routine documentation is insensitive to temporary, high-risk deviations in storage conditions [23,24].

This investigation contributes valuable, albeit inconclusive, evidence to the vaccine safety landscape in Timor-Leste. Injection-site abscess following hepatitis B vaccination appears to be a rare event, which inherently limits the power of retrospective studies to explore potential factors associated with abscess occurrence during an AEFI investigation. The findings underscore the critical need for robust AEFI surveillance systems capable of detecting signals and triggering timely investigations, as well as for prospective study designs that can capture exposures more accurately and with larger sample sizes.

## Strengths and Limitations

This study has several strengths. The matched case–control design helped control for potential confounding by vaccination facility and timing, reducing variability related to service delivery context. The investigation was embedded within the national AEFI response, enhancing its programmatic relevance. Data collection incorporated both caregiver interviews and facility-level assessments, providing a multi-source perspective.

Several important limitations should be acknowledged. First, the sample size was very small (12 matched pairs), which resulted in substantially limited statistical power. This is reflected in the wide confidence intervals surrounding all association estimates and the consequent inability to detect even moderate risk factors, should they exist. The small number of discordant pairs rendered the analysis particularly susceptible to instability. Given the high degree of instability in all inferential estimates, this study is unable to reliably confirm or refute any specific risk factor. Therefore, its primary utility lies in hypothesis generation and reinforcing the importance of safe practices.

Second, the retrospective design introduces the potential for recall and information bias. Exposures such as antiseptic use and vial handling were assessed approximately one year after the vaccination event and may be subject to inaccurate recall or social desirability bias.

Third, the analysis was constrained by limited variability in several key exposures. Variables with no discordant pairs (e.g., cold chain indicators) had to be excluded entirely. Given the small sample size, the analysis was limited to bivariate comparisons, and no multivariable modeling was performed to avoid overfitting and unreliable estimates.

Fourth, the diagnosis of injection-site abscess was based on clinical assessment without microbiological confirmation, which could lead to outcome misclassification between infectious abscesses and sterile inflammatory reactions.

Finally, reliance on routine AEFI surveillance for case identification means that underreporting cannot be excluded. The study sample may not fully represent all events that occurred during the study period.

Given these limitations, the findings should be interpreted as exploratory and hypothesis-generating, not as definitive evidence of causal relationships. The primary value of this study lies in highlighting the challenges of investigating rare adverse events and reinforcing the importance of standard safe practices.

## Conclusion

In this matched case-control study of injection-site abscess following hepatitis B birth-dose vaccination in Timor-Leste, no infant-related, provider-related, or vaccine-handling factors were statistically significantly associated with abscess occurrence in the primary matched analysis. These findings do not rule out the possibility that programmatic factors contribute to abscess formation. On the contrary, they reinforce the importance of maintaining rigorous adherence to safe immunization practices, including aseptic technique, proper skin preparation, and strict compliance with WHO multidose vial policies [21,22,25]. Strengthening AEFI surveillance systems to improve reporting completeness and analytical capacity remains essential for the early detection and robust investigation of rare vaccine-related adverse events.

Future research should prioritize prospective designs with larger sample sizes, standardized exposure assessment, and microbiological confirmation of outcomes to reliably explore potential factors associated with abscess occurrence during an AEFI investigation and inform targeted interventions. Ensuring the safety of routine immunization services is fundamental to sustaining public confidence in vaccination programs and protecting child health.

## Supporting information

S1 Data Raw data for the matched case-control study of risk factors for abscess development following hepatitis B vaccination in children in Timor-Leste, 2024. The dataset includes de-identified information for 12 case-control pairs, including infant demographics, birth history, vaccination procedures, provider practices, vaccine handling, cold chain maintenance, and outcome status.
(XLSX)

S2 Data Raw dataset for the matched case-control study (SPSS format). This file contains the same dataset as S1 Data, provided in IBM SPSS format (compressed as a zip file) for users of statistical software.
(RAR)
