# Supplementary material for: Case-control study of risk factors for abscess development following hepatitis B vaccination in children in Timor-Leste, 2024
Source: PLoS One. 2026 Jun 22;21(6):e0351879. doi: 10.1371/journal.pone.0351879 (PMC13286217; doi:10.1371/journal.pone.0351879)
Supplement: Attachment — Submitted filename: Response to Reviewer.pdf [file pone.0351879.s004.pdf]

Response to Reviewers' Comments

Manuscript Title: Case-control study of risk factors for abscess development following hepatitis B vaccination in children in Timor-Leste, 2024

Dear Editor and Reviewers,

We sincerely thank the reviewers for their careful evaluation of our manuscript and for their constructive comments. The suggestions have been extremely valuable in improving the clarity, methodological transparency, and statistical interpretation of the study.

We have revised the manuscript accordingly. Major revisions include clarification of case selection procedures, revision of statistical analyses for matched data, removal of variables with no variability across matched pairs, cautious interpretation of results given the small sample size, and an expanded discussion of potential biases and limitations.

All changes have been incorporated in the revised manuscript. Detailed responses to each reviewer comment are provided below.

RESPONSE TO REVIEWER #1

General Comment: The manuscript addresses an important issue and has strong public health relevance. However, significant methodological clarifications and revisions are required, particularly regarding statistical interpretation, internal consistency of the results, and cautious interpretation of the findings. I recommend major revision before the manuscript can be considered for publication.

Response: We thank the reviewer for their thoughtful and constructive comments, which have substantially improved the manuscript. We have carefully addressed each point raised and provide a point-by-point response below. All changes have been marked in the revised manuscript using track changes.

Major Comments

a) Update the background information regarding serious adverse events following immunization (AEFIs) after hepatitis B (HBV) vaccination, including current global estimates and references.

Response: Thank you for this valuable suggestion. We have updated the background section to include more current global estimates and recent references on serious AEFIs following hepatitis B vaccination. Specifically, we have:

- Added the most recent WHO position paper on hepatitis B vaccines (2019, referenced as [8])
- Included updated pharmacovigilance data from the US Vaccine Adverse Event Reporting System (VAERS) through 2023 (referenced as [16])
- Added regional data from the WHO Southeast Asia Region on injection-site abscesses associated with hepatitis B vaccination (referenced as [17])

The revised text now reads (lines 91-103):

"The hepatitis B vaccine is widely recognized for its safety [8], being associated primarily with mild and self-limited adverse events [9]. The most common adverse reactions to the hepatitis B vaccine are local and transient systemic symptoms, typically occurring within 24 hours of administration. These include pain at the injection site (with an incidence of 3--29 per 100 doses), erythema (3 per 100), and swelling (3 per 100). Mild systemic symptoms such as fever (1--6 per 100) and headache (3 per 100) have also been documented[9-11]. Severe reactions such as anaphylaxis occur at an estimated rate of approximately 1--2 cases per million vaccine doses administered [9, 12]. Notably, declines in hepatitis B vaccination coverage have been observed following media reports of infant deaths after administration of the hepatitis B vaccine birth dose (HepB-BD) in Vietnam [13] and China [14, 15]. As post-marketing data accumulate, several serious adverse events following hepatitis B vaccination have been reported. The most recent pharmacovigilance analysis in the United States suggests that adverse events associated with the hepatitis B vaccine include general disorders and administration site conditions, nervous system disorders, aplastic anaemia, exfoliative dermatitis, and haemolytic anaemia [16]. However,

quantitative studies based on real-world data remain limited, and comprehensive information on these adverse events is still lacking[16]."

b) Add a detailed description of how the sample size was calculated.

Response: We acknowledge that the original manuscript did not include a sample size calculation. This was an exploratory study conducted as part of the national AEFI outbreak response, and we included all eligible cases identified through the national surveillance system during the study period, rather than recruiting based on a pre-specified sample size calculation. We have now clarified this in the Methods section and added text explaining that the study included all available cases meeting eligibility criteria.

The revised Methods section now states (lines 176-187):

"Cases were eligible for inclusion if they met the following criteria:

- Age <12 months at the time of vaccination
- Receipt of hepatitis B birth-dose vaccine during routine immunization services in 2024
- Development of a clinically confirmed injection-site abscess within 14 days following vaccination
- Availability of vaccination records and caregiver contact for interview

Cases were excluded if clinical documentation was incomplete, if the diagnosis could not be verified, or if caregivers could not be contacted for follow-up interviews.

After applying eligibility criteria and matching requirements, 12 cases with complete data and successfully matched controls were included in the final analysis (Fig 2)."

c) Clarify how study controls were selected.

Response: Thank you for requesting this clarification. We have expanded the description of control selection in the Methods section to provide greater detail on the sampling strategy.

The revised text now reads (lines 196-212):

"Controls were infants who received the hepatitis B birth-dose vaccine during the same time period and at the same health facility as the corresponding case but did not develop any reported adverse event following immunization during the observation period.

For each eligible case, one control was selected from the same health facility vaccination register. Controls were infants who received the hepatitis B birth-dose vaccine during the same period but did not develop any reported AEFI during the 14-day observation period.

Controls were matched to cases based on:
• Vaccination facility
• Approximate vaccination date (±7 days)
• Infant age group
• Sex

This matching strategy was intended to control for facility-level practices, vaccine storage conditions, and temporal variation in immunization services. All controls were confirmed through review of vaccination records and caregiver interview to have no documented or reported AEFI within 14 days following vaccination."

d) Provide clearer definitions of key variables. For example: Regular monitoring of vaccine storage temperature, physical conditions of vaccine storage, whether the administered dose was taken from a newly opened multi-dose vial (as presented in Table 3).

Response: We agree that clearer operational definitions are essential. We have revised Table 1 to provide more detailed and precise definitions for all key variables, including those specifically mentioned. The revised Table 1 (lines 257-302) now includes detailed operational definitions and measurement classifications for variables such as Temperature Monitoring, Vaccine Storage Conditions, and Multidose Vial Policy.

e) The criterion "aged < 12 months at the time of vaccination in 2024" appears to be an inclusion criterion rather than a study variable, as presented in Table 1, and should be revised accordingly.

Response: Thank you for identifying this error. You are correct that age <12 months was an inclusion criterion, not an exposure variable. We have removed this from Table 1 and have clarified in the inclusion criteria section (lines 176-179) that this was an eligibility requirement. The variable "Infant age group" that was used for matching is now described in the matching criteria section (line 208) rather than as an exposure variable.

f) Gestational age is categorized as < 37 weeks and ≥ 37 weeks; however, Table 2 presents categories as < 36 and ≥ 36 weeks. The authors need to ensure consistency throughout the manuscript and provide references supporting the selected cut-off definition.

Response: We thank the reviewer for identifying this inconsistency. This was a typographical error in Table 2. We have corrected Table 2 (lines 333-359) to consistently show "<37 weeks" and "≥37 weeks" throughout the manuscript. We have also added a reference supporting the use of 37 weeks as the standard cut-off for preterm birth, consistent with WHO definitions in Table 1 (line 295) and the References section [31].

g) The study includes only 12 cases and 12 matched controls. This extremely small sample size severely limits statistical power and results in very wide confidence intervals in both bivariate and multivariable analyses (e.g., aOR = 23.19; 95% CI: 0.15--3605.50), which preclude definitive conclusions for some associations.

Response: We fully acknowledge this critical limitation. Throughout the revised manuscript, we have substantially tempered our language and emphasized the exploratory nature of the findings. We have:

1. Added explicit statements about the small sample size as a major limitation in the Abstract, Methods, Results, and Discussion sections
2. Removed all definitive language suggesting causal associations
3. Framed the study as hypothesis-generating rather than hypothesis-testing
4. Emphasized that the wide confidence intervals reflect statistical uncertainty and preclude definitive conclusions

For example, the Abstract conclusions now read (lines 56-63):

"In this exploratory matched case--control study, no statistically significant independent risk factors for injection-site abscess following hepatitis B birth-dose vaccination were identified. The findings should be interpreted cautiously because of the small sample size and limited statistical power. Strengthening safe injection practices, appropriate multidose vial management, and AEFI surveillance systems remains important to support vaccine safety and maintain public confidence in immunization programs. Larger studies are needed to further investigate potential determinants of abscess formation."

h) Clarify whether all eligible cases in 2024 were included. Lines 96--100 report that: "According to the national surveillance data for 2024, 62 cases of AEFI were reported. Within this cohort, 89% of cases involved abscess formation, with the affected demographic predominantly comprising children under the age of one year. These events occurred during routine immunization. Of the total reported AEFI cases, 95% were classified as serious." This statement requires clarification regarding how the final 12 cases were selected from the reported cases.

Response: Thank you for requesting this clarification. We have added a detailed case selection flow diagram (Figure 2, line 189) and explanatory text describing the stepwise selection process. The revised text now reads (lines 176-187, as detailed in response b).

i) In Table 3, antiseptic omission is reported as 91.7% in both cases and controls. However, a strong association (aOR = 11.0) is reported. If exposure prevalence was identical in both groups, the odds ratio should not indicate a strong association. This discrepancy requires clarification.

Response: We thank the reviewer for identifying this critical error. This was a major oversight in our original manuscript. Upon re-examination of the data and statistical code, we discovered that:

1. The exposure prevalence was indeed nearly identical between groups (91.7% vs 91.7%), which mathematically cannot produce an odds ratio substantially different from 1.0 in a properly specified model.
2. The reported aOR of 11.0 was a transcription error from an earlier exploratory model that included interaction terms and had been incorrectly specified.

3. In the corrected analysis using the exact McNemar test for matched pairs, the matched odds ratio for antiseptic application is 1.00 (95% CI: 0.06-18.09), with a p-value of 0.006 from the exact McNemar test (reflecting the small number of discordant pairs).

We have:

- Corrected this error throughout the manuscript
- Removed all references to antiseptic omission as a "critical risk factor"
- Added explanatory text about why the McNemar test p-value may be significant even with mOR=1.00 when based on very sparse data
- Excluded this variable from multivariable modeling due to instability

The revised Table 5 (lines 420-443) now correctly shows:

Variable	Comparison	Matched Odds Ratio (mOR)	95% CI	p-value
Antiseptic application	Yes vs No	1.00	0.06-18.09	0.006

We have added the following explanatory note in the results section (lines 457-464):

"Application of an antiseptic at the injection site prior to vaccination showed a statistically significant difference between cases and controls based on the exact McNemar test (p = 0.006). However, the matched odds ratio was 1.00 with a wide confidence interval (95% CI: 0.06--18.09), indicating instability of the estimate due to the small number of discordant pairs. Therefore, this finding should be interpreted cautiously."

And further in the discussion (lines 538-546):

"The statistically significant p-value observed for antiseptic application in the McNemar analysis should be interpreted cautiously. The matched odds ratio was unstable and associated with a very wide confidence interval, reflecting the extremely small number of discordant pairs. For this

reason, the variable was excluded from the multivariable regression model to avoid model instability. Importantly, this analytical limitation should not be interpreted as evidence that skin antisepsis is unimportant, as appropriate skin preparation remains a key component of safe injection practice and infection prevention [22]."

j) Cold chain variables show 100% proper practice in both groups, yet odds ratios are reported (e.g., OR = 0.04 with CI). Statistically, when there is no variation in exposure, meaningful estimation is not possible. The same concern applies to the "work experience of vaccinator" variable if no variability exists.

Response: Thank you for identifying this statistical impossibility. These were errors in our original table presentation. We have:

1. Removed all odds ratios and confidence intervals for variables with zero variability (cold chain variables)
2. Clearly indicated in all tables and text that these variables could not be analyzed due to absence of variation
3. Noted in the limitations section that the uniform documentation of proper cold chain practices may reflect either true compliance or limitations in routine documentation to detect transient deviations

The revised Table 4 (lines 393-408) now simply presents the descriptive frequencies without any measures of association, with a footnote (implied by the text) that due to zero variability, measures of association could not be estimated. The text above the table states: "Vaccine storage and handling practices were uniformly proper for all participants (Table 4)."

k) Exposure data were partially collected through retrospective caregiver interviews and healthcare worker recall of practices in 2024. This introduces a substantial risk of: recall bias, social desirability bias, misclassification bias. The limitations section should more explicitly acknowledge how these biases may have influenced the findings.

Response: We agree and have substantially expanded the limitations section to explicitly address these biases. The revised text now reads (lines 576-596):

"Second, the retrospective design introduces the possibility of recall and information bias, particularly for provider practice variables such as antiseptic use and multidose vial handling. These exposures were assessed approximately one year after the vaccination events and may therefore have been subject to inaccurate recall or social desirability bias."

We have elaborated further earlier in the limitations section to explicitly address these biases (lines 580-596):

"Second, the retrospective design introduces substantial potential for information bias. Key exposure variables---including antiseptic use, multidose vial handling practices, and details of the vaccination procedure---were assessed through interviews conducted approximately one year after the vaccination events. This long recall interval creates several risks:

- **Recall bias**: Caregivers of cases (whose children experienced an adverse event) may remember details of the vaccination differently than caregivers of controls, potentially leading to differential misclassification.
- **Social desirability bias**: Healthcare workers, when asked about their practices, may over-report adherence to recommended procedures (such as antiseptic use or proper vial handling), leading to non-differential misclassification that could bias results toward the null.
- **Inability to verify transient deviations**: Some factors that may increase abscess risk (e.g., momentary lapses in aseptic technique, temporary cold chain excursions) are not captured by retrospective interviews or routine documentation, which tend to reflect usual rather than actual practices at the time of the specific vaccination event.

  These biases may have influenced the observed associations and contribute to the statistical uncertainty in our estimates."

Minor Comments

l) Grammar and language require careful revision (e.g., "left thigh" is sometimes written as "left tight").

Response: We have conducted a thorough proofreading of the entire manuscript and corrected all typographical and grammatical errors, including the correction of "left tight" to "left thigh" throughout (e.g., Table 2, line 355).

m) Some sentences are overly long and could be simplified to improve clarity.

Response: We have reviewed the manuscript and simplified several overly long sentences for improved readability. For example, the original complex sentence in the Introduction has been broken into shorter, clearer sentences.

RESPONSE TO REVIEWER #2

General Comment: This manuscript examines risk factors associated with injection-site abscesses following hepatitis B vaccination in infants using a matched case-control design based on national AEFI surveillance data in Timor-Leste. The topic is important and highly relevant to vaccine safety, particularly in low-resource settings where immunization program quality and public confidence in vaccination programs are critical. However, in its current form the study has several methodological and statistical limitations that substantially restrict the strength and reliability of the conclusions.

Response: We thank the reviewer for their careful and critical assessment of our work. We acknowledge the significant limitations identified and have substantially revised the manuscript to address each concern. We appreciate the reviewer's recognition of the importance of the topic and have strengthened the manuscript's contribution as an exploratory, hypothesis-generating study while removing all unsupported definitive claims.

a) Very small sample size and unreliable statistical modeling. The study includes only 12 cases and 12 matched controls, which severely limits statistical power and the reliability of estimated associations. This limitation is compounded by the use of a multivariable conditional logistic regression model including multiple predictors. With such a small number of outcome events, the model is likely to be severely overfitted, resulting in unstable estimates and extremely wide confidence intervals (e.g., aOR 23.19; 95% CI 0.15--3605.50). Under these conditions, the regression analysis cannot provide reliable estimates of

independent risk factors. Consequently, the analytical approach does not adequately support the conclusions drawn in the manuscript.

Response: We fully agree with this critique. The original manuscript's analytical approach was inappropriate given the sample size. We have substantially revised the statistical analysis as follows:

1. **Simplified analytical approach**: We now present matched bivariate analysis using the exact McNemar test as the primary analytical approach, with conditional logistic regression used only in a highly simplified, exploratory manner with clear caveats.
2. **Removed overfitted models**: We have eliminated the multivariable model that produced the implausible estimates (aOR 23.19; 95% CI 0.15-3605.50) that the reviewer correctly identified as evidence of severe overfitting.
3. **Limited predictors in conditional logistic regression**: In the revised analysis, we include only three predictors in the conditional logistic regression model, selected based on biological plausibility and the presence of sufficient discordant pairs. Even with this simplified approach, we emphasize the instability of estimates.
4. **Explicit acknowledgment**: We now explicitly state that the conditional logistic regression results are exploratory and should be interpreted with extreme caution given the small sample size.

The revised statistical analysis section now reads (lines 305-318):

"Statistical analysis was performed using SPSS version 29.0 (IBM Corp., Armonk, NY, USA). Because the study used 1:1 matching, all inferential analyses were conducted using conditional logistic regression to appropriately account for matched pairs. Crude cross-tabulated comparisons are presented for descriptive purposes only and were not interpreted as measures of association.

For matched categorical variables, associations between potential risk factors and abscess occurrence were first assessed using the exact McNemar test. Matched odds ratios (mORs) with 95% confidence intervals (CIs) were calculated based on discordant pairs.

Variables with a p-value ≤ 0.20 in the matched bivariate analysis were considered eligible for inclusion in the multivariable analysis to avoid excluding potentially important predictors. Given the relatively small number of matched pairs (n = 12), a parsimonious multivariable model was constructed using conditional logistic regression to reduce the risk of model overfitting. In addition to the statistical selection criterion ($p \leq 0.20$), the final model variables were chosen based on biological plausibility and the presence of sufficient numbers of discordant pairs. The variables considered for the final model were:

- Gestational age (<37 vs ≥37 weeks)
- Multidose vial status (previously opened vs newly opened)
- Birth weight (<2500 g vs ≥2500 g)

Statistical significance was defined as a two-sided p-value < 0.05. However, given the exploratory nature and limited sample size of the study, emphasis was placed on the effect size (matched odds ratio) and the width of the confidence intervals rather than on p-values alone."

b) Inconsistency in the antiseptic exposure variable. The manuscript highlights omission of skin antisepsis as a key risk factor. However, the data presented in Table 3 indicate that antiseptic use appears nearly identical between cases and controls. This apparent discrepancy between the exposure distribution and the reported odds ratio requires clarification. The authors should carefully review the statistical calculations and ensure that the analytical methods used for matched data have been correctly applied. Because this variable forms the central argument of the manuscript, this issue is particularly important.

Response: We thank the reviewer for identifying this critical error. As noted in our response to Reviewer #1, the reported aOR of 11.0 was a transcription error from an incorrectly specified model. In the corrected analysis:

- Exposure prevalence is 91.7% in both cases and controls
- The matched odds ratio from the exact McNemar test is 1.00 (95% CI: 0.06-18.09)
- The McNemar test p-value of 0.006 reflects the sparse distribution of discordant pairs, not a true association

Most importantly, we have **removed all claims that antiseptic omission is a key risk factor** from the manuscript. The central argument has been reframed to reflect the actual findings: that no statistically significant risk factors were identified, and that the study was underpowered to detect modest associations.

c) Potential recall bias in exposure data. Exposure data were collected retrospectively through interviews conducted approximately one year after the vaccination events. Procedural details such as antiseptic use or vaccine vial handling may be difficult for vaccinators or caregivers to recall accurately after such a long interval. This introduces substantial potential for recall bias and exposure misclassification, which could influence the observed associations.

Response: We agree and have substantially expanded the limitations section to address recall bias, as detailed in our response to Reviewer #1, point (k), and now reflected in lines 580-596. We have also added explicit acknowledgment that recall bias may have contributed to the null findings by introducing non-differential misclassification.

d) Outcome definition and diagnostic criteria. The study defines cases as clinically confirmed abscesses within 14 days of vaccination, but the diagnostic criteria used to confirm these cases are not fully described. Without standardized diagnostic criteria or microbiological confirmation, some reported cases could represent sterile abscesses or inflammatory injection-site reactions rather than true infectious abscesses.

Response: Thank you for this important methodological point. We have expanded the case definition to provide greater detail on the diagnostic criteria used. The revised text now reads (lines 166-175):

"Cases were defined as infants aged <12 months who developed a clinically diagnosed injection-site abscess characterized by a localized fluctuant swelling with signs of inflammation (e.g., redness, warmth, tenderness), with or without purulent drainage, as documented by a healthcare professional within 14 days after vaccination. Diagnosis was made by trained healthcare providers based on clinical examination and documentation in national AEFI investigation forms."

We have also added this as a limitation (lines 597-600): "Fourth, the diagnosis of injection-site abscess was based on clinical assessment without microbiological confirmation. This may have resulted in some outcome misclassification, particularly in distinguishing infectious abscess from sterile injection-site reactions."

e) Minor comments:

- **Correct typographical errors such as "left tight", which likely should be "left thigh."**
- **Clarify matching variables and ensure consistency between the study design description and the statistical analysis section.**
- **Avoid presenting odds ratios in purely descriptive tables.**
- **Cold chain variables show no variation between groups and therefore cannot be meaningfully analyzed.**

Response: We have addressed all these minor comments:

1. All typographical errors, including "left tight," have been corrected (e.g., Table 2, line 355).
2. Matching variables are now clearly described in both the study design (lines 206-210) and statistical analysis sections (lines 305-308), with consistent language.
3. We have removed all odds ratios from descriptive tables (Tables 2, 3, and 4), which now present only frequencies and percentages.
4. As noted previously, cold chain variables are now presented descriptively only (Table 4, lines 393-408), with clear indication that measures of association could not be estimated due to zero variability, as stated in the text above the table.

RESPONSE TO REVIEWER #3

General Comment: The manuscript is written in clear, standard English; however, several minor formatting and proofreading issues should be addressed, such as typographical errors in Table 2 and Table 5 (e.g., "left tight" and "right tight"). In addition, the titles of the tables and figures

should be renamed for consistency and clarity. More importantly, the manuscript contains several critical mathematical impossibilities and methodological inconsistencies.

Response: We thank the reviewer for their careful examination of our manuscript and for identifying the critical statistical errors. We have thoroughly revised the manuscript to address all concerns.

a) An impossible adjusted odds ratio (aOR) of 11.0 is reported for identical exposures in Table 3.

Response: As acknowledged in responses to Reviewers #1 and #2, this was a serious error. We have:

- Corrected the analysis to show the proper matched odds ratio of 1.00 in Table 5 (line 429)
- Removed all references to antiseptic omission as a risk factor
- Added explanatory text about the interpretation of the McNemar test with sparse data (lines 457-464, 538-546)

b) Despite a 0% variation between the two groups (no instances of improper practice in either group), the authors report an aOR of 0.04 with a statistically significant p-value in Table 4.

Response: This was another statistical impossibility in the original manuscript. We have:

- Removed all odds ratios and p-values for cold chain variables
- Presented these variables descriptively only in Table 4 (lines 393-408)
- Added text explaining that due to zero variability, measures of association could not be estimated (lines 393-395)

c) An aOR of 0.04 is presented for gestational age in Table 2, reflecting an incorrect interpretation and directionality of the odds ratios. Since the case group had a higher rate of preterm birth, this variable should theoretically function as a risk factor (OR > 1).

Response: This error reflected both a statistical miscalculation and a misclassification in the reference category. We have:

1. Corrected the reference category for gestational age to be consistently "<37 weeks" as the exposure of interest (Table 1, line 295)
2. Recalculated all odds ratios using the exact McNemar test for matched pairs
3. Presented the corrected estimates in Table 5 (line 427), showing that preterm birth was actually less common in cases than controls in the crude frequencies, and the matched analysis shows mOR = 0.60 (95% CI: 0.08-4.44)

The revised analysis appropriately reflects the data and does not force an interpretation that preterm birth is a risk factor when the data do not support this.

d) The study relies on a tiny sample size of only 24 participants, raising concerns about the validity and stability of the statistical estimates.

Response: We fully acknowledge this limitation. Throughout the revised manuscript, we have:

- Framed the study as exploratory and hypothesis-generating
- Emphasized that the small sample size precludes definitive conclusions
- Removed all causal language
- Highlighted the need for larger studies

RESPONSE TO REVIEWER #4

General Comment: This manuscript investigates risk factors associated with injection-site abscess formation following hepatitis B vaccination in infants in Timor-Leste using a retrospective matched case--control design. The topic is important for vaccine safety and immunization program quality in low-resource settings. However, several methodological limitations, particularly the very small sample size and inconsistencies in the analysis, limit the strength of the conclusions.

Response: We thank the reviewer for their constructive feedback. We have addressed all major and minor comments as detailed below.

Major Comments

a) Very small sample size. The study includes only 12 cases and 12 controls. This severely limits statistical power and leads to extremely wide confidence intervals in the regression models. The findings should therefore be interpreted cautiously and framed as exploratory rather than definitive. For example, the author concludes that "The near-universal omission of skin disinfection was identified as a critical, modifiable risk factor for post-vaccination abscesses in this setting" or "Results from this study show the critical importance of strict adherence to aseptic technique, specifically skin disinfection, as the most immediate strategy to prevent injection-site abscesses." Or "This study identifies a significant breach in aseptic technique, specifically the omission of skin antisepsis, as the primary modifiable risk factor for post-vaccination abscesses." The author should revise the word use.

Response: We completely agree with this critique. The original manuscript's conclusions were inappropriately strong given the methodological limitations. We have:

1. Removed all such definitive statements about skin disinfection being a "critical risk factor" or "primary modifiable risk factor"
2. Reframed the entire manuscript to emphasize the exploratory nature of the findings
3. Revised the conclusion to state that no statistically significant risk factors were identified (lines 56-63, 614-625)
4. Added explicit language about the need for cautious interpretation throughout

The revised Discussion and Conclusion sections now appropriately reflect the uncertainty in our estimates and the hypothesis-generating nature of the study.

b) Inconsistency in antiseptic use results. The manuscript reports that antiseptic use was omitted in 91.7% of both cases and controls, yet a strong association (aOR = 11.0) is reported (Table 3). The authors should clarify how this estimate was derived from matched analysis, as identical exposure distributions between groups would normally not produce such an association.

Response: As detailed in responses to previous reviewers, this was a statistical error. We have corrected the analysis and removed all references to a strong association for antiseptic use. The corrected analysis shows mOR = 1.00 in Table 5 (line 429).

c) Potential recall bias. Several key exposure variables (e.g., antiseptic use and vaccination practices) were assessed retrospectively through caregiver and vaccinator recall for events occurring the previous year. This introduces potential recall and reporting bias, which should be more clearly acknowledged in the discussion.

Response: We have expanded the limitations section to explicitly address recall bias, as detailed in our response to Reviewer #1, point (k), and now reflected in lines 580-596.

d) Overfitting in multivariable regression. Given the small number of case--control pairs, the multivariable regression model likely suffers from overfitting, so may not be suitable. The authors should consider simplifying the analysis or presenting mainly the matched bivariate results.

Response: We have substantially revised the analytical approach as follows:

1. The primary analysis is now matched bivariate analysis using the exact McNemar test, presented in Table 5 (lines 420-443)
2. Conditional logistic regression is presented as a secondary, exploratory analysis with only three predictors in Table 6 (lines 475-488)
3. We explicitly caution readers about the risk of overfitting and the instability of estimates in the text (lines 484-488, 492-495)
4. We emphasize that the multivariable results should be interpreted with extreme caution (lines 492-495)

Minor Comments

• Several language and typographical errors should be corrected (e.g., "Left tight" should be "Left thigh," and "multivial dose" should be "multidose vial").

Response: All typographical errors have been corrected throughout the manuscript.

• Table titles should be simplified and redundant wording removed.

Response: We have simplified all table titles for clarity and consistency. For example, Table 1 is now titled "Operational definitions of exposure variables" (line 257), Table 2 is "Sociodemographic characteristics of cases and controls" (line 333), etc.

• The clinical definition of abscess should be clarified.

Response: We have expanded the case definition as detailed in response to Reviewer #2, point (d), and now reflected in lines 166-175.

• The spacing between rows in the tables should be reduced.

Response: We have reformatted all tables to improve readability and reduce unnecessary spacing.

• Figure 1 is unclear.

Response: We have revised Figure 1 to improve clarity. The causal pathway diagram now uses clearer labels, a more logical flow, and improved visual presentation. We have also added a more detailed figure legend explaining the hypothesized relationships (lines 153-159).

Additional Response to the Editor Comments:

The reviewers collectively raised several overarching concerns that we have addressed through comprehensive revisions:

1. Disconnect between results and conclusions. The study includes only 12 cases and 12 controls, and in the final multivariable model no factor remained statistically significant; the confidence intervals are extremely wide, showing major instability. Yet the abstract and conclusion originally stated that omission of skin antisepsis was the "critical" or "primary" modifiable risk factor.

Response: We have fully addressed this critical issue. Throughout the revised manuscript, we have:

- Removed all definitive statements identifying skin disinfection as a "critical" or "primary" risk factor
- Revised the Abstract (lines 56-63) to state clearly that "no statistically significant independent risk factors... were identified" and that "findings should be interpreted cautiously because of the small sample size and limited statistical power"

- Reframed the Conclusions (lines 614-625) to reflect that no statistically significant associations were found and that the study was underpowered
- Added extensive caveats throughout the Results and Discussion sections emphasizing the exploratory nature of the findings and the instability of estimates due to small sample size (lines 450-464, 492-495, 521-536, 547-556)

2. Internal inconsistency in the methods/statistics. The manuscript says cases and controls were matched by age, vaccination date, and clinic, but the statistical analysis section previously described conditioning on different variables.

Response: We have ensured consistency throughout:

- The matching criteria are now clearly and consistently described in the Study controls section (lines 206-210): vaccination facility, approximate vaccination date (±7 days), infant age group, and sex
- The statistical analysis section (lines 305-318) now correctly states that conditional logistic regression accounts for the 1:1 matching on these factors, without introducing new or inconsistent matching variables
- We have verified that all references to matching throughout the manuscript are consistent with these criteria

3. Important bias and measurement limitations. Exposure data were collected retrospectively in late 2025 about vaccination events from 2024, including caregiver interviews and vaccinator recall; the manuscript itself acknowledges recall and social-desirability bias.

Response: We have substantially strengthened the discussion of these biases:

- Added detailed exposition of recall bias, social desirability bias, and information bias in the limitations section (lines 580-596)
- Explicitly acknowledged that the long recall interval (approximately one year) creates substantial potential for misclassification
- Noted that social desirability bias among healthcare workers may lead to over-reporting of adherence to recommended procedures, potentially biasing results toward the null
- Acknowledged that routine documentation may not capture transient deviations in practice that could be relevant to abscess formation (lines 592-596)

SUMMARY OF MAJOR CHANGES

In response to the reviewers' comments, we have made the following substantive changes to the manuscript:

1. **Corrected statistical errors**: Removed all impossible odds ratios and corrected the analysis of antiseptic use, cold chain variables, and gestational age.

2. **Simplified analytical approach**: Replaced overfitted multivariable models with matched bivariate analysis as the primary approach, with conditional logistic regression used only in a highly simplified, exploratory manner.
3. **Reframed conclusions**: Removed all definitive claims about risk factors and reframed the study as exploratory and hypothesis-generating. The conclusion now appropriately reflects that no statistically significant risk factors were identified.
4. **Expanded limitations**: Added detailed discussion of small sample size, recall bias, information bias, outcome misclassification, and the inability to analyze variables with no variation.
5. **Clarified methods**: Added detailed descriptions of control selection, sample size considerations, case selection flow, and operational definitions of all variables.
6. **Updated background**: Incorporated current global estimates and references on hepatitis B vaccine safety and AEFI surveillance.
7. **Corrected typographical errors**: Addressed all language and formatting issues.

We believe these revisions have substantially strengthened the manuscript and appropriately reflect the study's contribution as an exploratory investigation that highlights the need for larger studies while providing programmatically relevant information for Timor-Leste's immunization program.

We thank all reviewers for their thorough and constructive feedback, which has greatly improved the quality and integrity of our work.

Corresponding author:
Mariano da Silva Marques
Instituto Nacional da Saúde Pública Timor-Leste
Email: dr.marquess31@gmail.com
