# Supplementary material for: Case-control study of risk factors for abscess development following hepatitis B vaccination in children in Timor-Leste, 2024
Source: PLoS One. 2026 Jun 22;21(6):e0351879. doi: 10.1371/journal.pone.0351879 (PMC13286217; doi:10.1371/journal.pone.0351879)
Supplement: Attachment — Submitted filename: Response to Reviewers.pdf [file pone.0351879.s005.pdf]

Response to Reviewers' Comments

Manuscript Title: Case-control study of risk factors for abscess development following hepatitis B vaccination in children in Timor-Leste, 2024

Dear Editor and Reviewers,

Thank you for the opportunity to revise our manuscript and for the thoughtful and constructive feedback provided during the review process. We have carefully considered all comments and have revised the manuscript accordingly. Below, we provide a point-by-point response to each comment. All changes have been made using track changes in the revised manuscript, and page and line numbers refer to the clean version of the revised document.

We believe these revisions have substantially strengthened the manuscript and hope it is now suitable for publication in PLOS ONE.

Response to Editor and Reviewers

Reviewer Comment 1: Recheck the antiseptic analysis from the raw matched-pair counts. This is the biggest issue. The authors should present the discordant pair counts explicitly and make sure the matched odds ratio, confidence interval, and McNemar p-value all come from the same data and method. If the mOR is truly 1.00, the p-value likely needs correction.

Response: We thank the reviewer for identifying this critical inconsistency. Upon re-examination, we confirmed that the discordant pairs for antiseptic application were indeed 1 and 1, which yields a matched odds ratio of 1.00 and an exact McNemar p-value of 1.000. The previously reported p-value of 0.006 was a transcription error. We have corrected this in Table 4 (page 17, line 278) and have verified that all other p-values in the table are accurate. We appreciate the reviewer's diligence in catching this error.

Reviewer Comment 2: Consider removing the conditional logistic regression entirely. With only 12 matched pairs, even a 3-variable model is still fragile. The paper would probably be stronger if it relied mainly on descriptive matched analysis and stated clearly that the study was underpowered for robust multivariable inference.

Response: We agree with the reviewer's assessment. We have removed the conditional logistic regression analysis entirely from the manuscript. The results now focus exclusively on the exact McNemar test for matched bivariate associations, which is more appropriate given the small sample size. We have also strengthened the language throughout the Discussion and Limitations sections to emphasize that the study was underpowered for robust multivariable inference and that findings should be interpreted as exploratory and hypothesis-generating. These revisions can be found in the Statistical Analysis section (page 13, lines 224), the Discussion (page 19, lines 302 and the Strengths and Limitations section (page 21, lines 350).

Reviewer Comment 3: Reframe the study aim slightly. Instead of "identify risk factors," use language like "explore potential factors associated with abscess occurrence during an AEFI investigation." That wording better matches what the data can support.

Response: We have revised the study aim throughout the manuscript to reflect the exploratory nature of this investigation. Specifically:

In the Abstract (page 2, lines 22–24), we now state: "This study aimed to explore potential factors associated with abscess formation to inform improvements in immunization practices."

In the Introduction (page 7, lines 130–132), we have revised the final sentence to read: "This study aimed to explore potential factors associated with abscess occurrence during an AEFI investigation following hepatitis B vaccination using multidose vials in children in Timor-Leste."

Reviewer Comment 4: Add a clearer selection flow from the surveillance pool to the final 12 pairs. The reviewers asked about how the final 12 were derived from the larger 2024 AEFI reports. The authors say they added a flow diagram, which is good, but this needs to be very explicit and easy to follow in the manuscript.

Response: We have added Figure 2 (page 10, lines 184–186), which provides a clear visual representation of the participant selection process. The flow diagram shows the stepwise derivation of the 12 matched pairs from the initial 62 AEFI reports, including the application of eligibility criteria and matching requirements. We have also expanded the accompanying text in the Study Cases section (page 9, lines 161–200) to describe the selection process in detail. We believe this fully addresses the reviewer's concern regarding transparency of the selection process.

Reviewer Comment 5: State very clearly that absence of significant association does not mean absence of risk. The revised paper is better on this point already, but it should say plainly that the study may have missed true associations because of low power, misclassification, and recall bias.

Response: We have further strengthened this important point. In the Discussion (page 19, lines 312–319), we have added a dedicated paragraph that explicitly states:

"Importantly, the absence of statistically significant associations in this study should not be interpreted as evidence of absence of risk. Given the very small number of matched pairs, this study had limited statistical power to detect anything other than very large effects. As a result, true associations between vaccination practices or recipient characteristics and abscess formation may have been missed. In addition, potential outcome misclassification and recall bias in the retrospective assessment of exposures could have further attenuated observed associations toward the null. The findings should therefore be interpreted as exploratory and hypothesis-generating rather than definitive."

Reviewer Comment 6: Move zero-variation variables to descriptive text or supplement. Since cold-chain variables could not be analytically estimated, keeping them minimal will tighten the paper.

Response: We agree with the reviewer's suggestion to streamline the manuscript. We have moved the cold-chain variables (temperature monitoring and vaccine storage conditions) out of the analytical table and now describe them only in the text. Specifically, we state in the Results section (page 16, lines 269–374):

"Vaccine storage and handling practices were uniformly documented as compliant across all participating facilities. For both cases and controls, temperature monitoring logs indicated regular documentation, and physical storage conditions met national cold chain requirements. As no variability was observed in these indicators, they were excluded from the matched analysis."

Additional Revisions

In addition to the changes requested by the reviewer, we have made the following minor revisions to improve manuscript clarity:

Table 4: We verified all discordant pair counts and p-values to ensure consistency with the exact McNemar test calculations. All values are now accurate and internally consistent (page 17, lines 278).

Strengths and Limitations: We added a brief statement acknowledging the potential for selection bias due to the matching requirements (page 21, lines 351–378). While not explicitly requested, we believe this addition further enhances transparency regarding the study's limitations.

**Reviewer Comment**	**Action Taken**	**Location (Page, Lines)**
Correct antiseptic p-value	Corrected from 0.006 to 1.000 in Table 4	Page 17, line 278
Remove conditional logistic regression	Removed entirely; analysis now focuses on exact McNemar test	-
Reframe study aim	Revised aim to exploratory language in Abstract and Introduction	Page 2, lines 22–24; Page 7, lines 130–132
Add clearer selection flow	Added Figure 2 and expanded text	Page 9, lines 161-200
Clarify absence of significance ≠ absence of risk	Added dedicated paragraph in Discussion	Page 19, lines 312–319
Move zero-variation variables	Described cold-chain variables in text only; excluded from analysis	Page 16, lines 270–274

Selection bias acknowledgment (additional)	Added statement in Strengths and Limitations	Page 21, lines 350–378

Conclusion

We are grateful for the reviewers' insightful comments, which have helped us substantially improve the clarity, rigor, and transparency of our manuscript. We believe the revised version now meets the high standards of PLOS ONE and respectfully submit it for your final consideration.

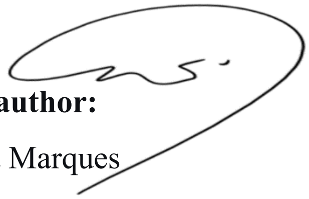

Corresponding author:
Mariano da Silva Marques
Instituto Nacional da Saúde Pública Timor-Leste
Email: dr.marquess31@gmail.com
