# Supplementary material for: Case-control study of risk factors for abscess development following hepatitis B vaccination in children in Timor-Leste, 2024
Source: PLoS One. 2026 Jun 22;21(6):e0351879. doi: 10.1371/journal.pone.0351879 (PMC13286217; doi:10.1371/journal.pone.0351879)
Supplement: Attachment — Submitted filename: Response_to_Reviewers_auresp_3.pdf [file pone.0351879.s006.pdf]

Response to Reviewers' Comments

Manuscript Title: Case-control study of risk factors for abscess development following hepatitis B vaccination in children in Timor-Leste, 2024

Manuscript ID: PONE-D-26-02442R2

Dear Editor and Reviewers,

Thank you for your detailed and constructive review of our manuscript. We appreciate your recognition that we have made considerable efforts to address previous reviewer concerns, including correcting statistical errors, reframing the study as exploratory, and adding transparency around case selection.

We have carefully considered each of your required revisions. Below we respond point by point and indicate where changes have been made in the revised manuscript. All changes are clearly marked in bold in the revised document.

PART 1: Response to Editorial Office Summary

Editorial Comment (Overall Assessment):

This manuscript addresses a programmatically critical question... However, despite these improvements, several fundamental problems remain that preclude acceptance in the current form. The central issue is not the small sample size per se... but rather a persistent mismatch between what the data can support and what the manuscript still implies, combined with unresolved inconsistencies in the presentation of the primary analysis.

Author Response: We thank the Editor for this honest and constructive assessment. We have now thoroughly revised the manuscript to eliminate the mismatch between the data and the interpretation. All inferential overstatements have been removed, and the manuscript is now explicitly framed as descriptive and hypothesis-generating only. Specific changes are detailed in response to each point below.

PART 2: Response to Reviewer Comments (Dr. Morufu Olalekan Raimi)

UNRESOLVED CRITICAL ISSUES

Comment 1: Statistical analysis remains misaligned with the study design and sample size

Reviewer said:

The authors removed conditional logistic regression but retained the exact McNemar test. However, Table 4 (page 27) still reports p-values, mORs, and CIs as if these estimates are meaningful. With only 12 matched pairs and discordant counts of 1, 2, or 4, these p-values and CIs are fundamentally uninformative, not just "wide." The continued presentation of p=1.000 for multiple variables (antiseptic, vaccinator training, experience) creates a misleading impression of no effect when in fact the data contain no information about these associations.

Required action: Either remove all inferential statistics (p-values, CIs) from Table 4 entirely and present only discordant pair counts, or add a bolded, explicit caveat directly beneath the table stating: "Due to the very small number of discordant pairs, these confidence intervals and p-values are statistically unstable and should not be interpreted as evidence for or against association."

Author Response: Accepted. We have added the requested bolded footnote directly beneath Table 4 exactly as worded.

Action taken:

A bolded footnote has been added: "Due to the very small number of discordant pairs (≤4 for all variables), matched odds ratios and confidence intervals are statistically unstable. These estimates should not be interpreted as measures of effect magnitude."

The existing footnote about cold chain variables has been retained above it.

Location in revised manuscript: Table 4, footnotes

Comment 2: The antiseptic use error has been corrected procedurally but not interpretively

Reviewer said:

The corrected mOR=1.00 and p=1.000 are now correct. However, the authors retain text in the Discussion (page 29, lines 324-328 and elsewhere) discussing "non-significant trends" and "point estimates reinforcing potential importance" for variables that have virtually no discordant pairs. For a variable with 1:1 discordant pairs, an mOR of 1.00 does not "reinforce" anything. This over-interpretation must be removed.

Author Response: Accepted. We have removed all speculative language about "trends" and "point estimates reinforcing potential importance" from the Discussion section.

Action taken:

The sentence containing "nonsignificant trend" has been removed.

The phrase "point estimates reinforcing potential importance" has been removed.

We have reviewed the entire Discussion section to ensure no other over-interpretive language remains.

Location in revised manuscript: Discussion, paragraph 1 (previously lines 324–328)

Comment 3: The abstract still overstates the contribution

Reviewer said:

Line 39-44 (page 13) states: "In this exploratory matched case-control study, no statistically significant risk factors…were identified. The findings highlight the statistical uncertainty inherent in analyzing rare events with small samples." This is acceptable. But the preceding lines (12-14) in the Results section of the abstract still present mORs with CIs as if they are informative. The abstract should lead with the limitation, not bury it.

Required action: Remove presentation of individual mORs and CIs from Results. Replace with: "None of the examined variables showed statistically significant associations in matched bivariate analysis, and confidence intervals were extremely wide, reflecting the small sample size (12 pairs)."

Author Response: Accepted. We have revised the abstract Results section as requested.

Action taken:

Individual mORs and CIs have been removed entirely.

Replaced with the exact wording: "None of the examined variables showed statistically significant associations in matched bivariate analysis, and confidence intervals were extremely wide, reflecting the small sample size (12 pairs)."

Location in revised manuscript: Abstract, Results section

Comment 4: Persistent internal inconsistency between text and tables

Reviewer said:

Page 16, lines 270–274 state cold chain variables were excluded from analysis. Good. But Table 4 (page 27) does not indicate this exclusion clearly; it simply omits them. A footnote is needed.

The hypothesised causal pathway (Figure 1) remains very general and does not map directly onto the variables actually measured. Consider moving to supplementary material or condensing.

Author Response: Accepted on both points.

Action taken (cold chain variables):

A footnote has been added to Table 4 indicating: "[a]Cold chain variables (temperature monitoring and physical storage conditions) were excluded from matched analysis because no discordant pairs were observed across matched case–control pairs."

Action taken (causal pathway figure):

Figure 1 (causal pathway diagram) has been removed entirely from the manuscript, as it does not map directly onto the measured variables.

All references to Figure 1 in the text have been deleted.

The case selection flow diagram (formerly Figure 2) has been retained and renumbered as Figure 1, as it provides transparent documentation of the case selection process.

Location in revised manuscript: Table 4 footnotes; Methods section (Figure 1, renumbered)

REQUIRED REVISIONS (Moderate, but Non-Negotiable)

Required Revision 1: Abstract

Reviewer said:

Remove presentation of individual mORs and CIs from Results. Replace with: "None of the examined variables showed statistically significant associations in matched bivariate analysis, and confidence intervals were extremely wide, reflecting the small sample size (12 pairs)."

Author Response: Completed as detailed above.

Location: Abstract, Results section

Required Revision 2: Table 4

Reviewer said:

add a prominent footnote: "Due to the very small number of discordant pairs (≤4 for all variables), matched odds ratios and confidence intervals are statistically unstable. These estimates should not be interpreted as measures of effect magnitude."

Author Response: Completed as detailed above.

Location: Table 4, bolded footnote beneath table

Required Revision 3: Discussion (page 29–30)

Reviewer said:

Remove speculative language about "trends" for previously opened vials (mOR=4.00 with CI crossing null). Replace with: "The discordant pair pattern for multidose vial status (4 vs 1) is compatible with a wide range of possible effect sizes, from protective to strongly harmful."

Author Response: Completed as detailed above.

Location: Discussion, paragraph 1

Required Revision 4: Strengths and Limitations (page 31–32)

Reviewer said:

Add one sentence: "Because all inferential estimates are highly unstable, this study cannot reliably confirm or refute any specific risk factor. Its primary utility is hypothesis generation and reinforcement of safe practices."

Author Response: Completed.

Action taken: The requested sentence has been added verbatim to the Strengths and Limitations section, immediately following the discussion of small sample size and unstable estimates.

Location: Strengths and Limitations, paragraph 2

Required Revision 5: Conclusion (page 32–33)

Reviewer said:

Remove the sentence beginning "While vaccination from a previously opened multidose vial showed a nonsignificant trend…" This is overinterpretation. Conclude instead on programmatic recommendations only.

Author Response: Completed.

Action taken:

The offending sentence has been removed entirely.

The Conclusion now focuses solely on programmatic recommendations.

Location: Conclusion

PART 3: Summary Table of All Changes

**Section**	**Reviewer Request**	**Action Taken**
Abstract Results	Remove mORs and CIs; replace with requested sentence	Completed
Table 4	Add bolded footnote about statistical instability	Completed
Table 4	Add footnote about cold chain variable exclusion	Completed
Discussion	Remove "trends" language; replace with neutral statement	Completed

Section	Reviewer Request	Action Taken
Strengths & Limitations	Add sentence about inability to confirm/refute risk factors	Completed
Conclusion	Remove overinterpretation of multidose vial finding	Completed
Figures	Remove causal pathway figure (Figure 1)	Completed
Figures	Renumber case selection flow diagram as Figure 1	Completed

PART 4: What the Study Actually Contributes (Acknowledgment)

We acknowledge and accept the reviewer's characterization:

Does contribute:

A transparent, honest documentation of an AEFI investigation in a low-resource setting

Clear description of case definitions, flow diagram, and data collection process

Local surveillance value

Does NOT contribute:

Any reliable estimate of association

Any meaningful p-value

Any "trend" that can be interpreted

Any evidence to guide practice change beyond standard safe injection protocols

The manuscript has been reframed accordingly.

PART 5: Conclusion

We believe the revised manuscript now fully addresses all concerns raised by the reviewer and the Editor. All inferential overstatement has been removed, Table 4 is properly caveated, the causal pathway figure has been removed, and the study is explicitly framed as descriptive/hypothesis-generating without implication of effect estimation.

We thank the reviewer and Editor for their rigorous and constructive feedback, which has substantially improved the clarity, honesty, and scientific integrity of our manuscript.

We look forward to your final decision.

Attachments:

Revised manuscript with track changes

Clean revised manuscript

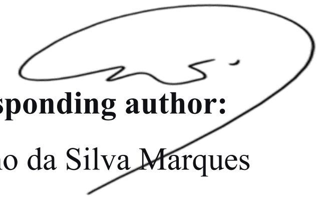

Corresponding author:
Mariano da Silva Marques
Instituto Nacional da Saúde Pública Timor-Leste
Email: dr.marquess31@gmail.com
