# Supplementary material for: Case-control study of risk factors for abscess development following hepatitis B vaccination in children in Timor-Leste, 2024
Source: PLoS One. 2026 Jun 22;21(6):e0351879. doi: 10.1371/journal.pone.0351879 (PMC13286217; doi:10.1371/journal.pone.0351879)
Supplement: Attachment — Submitted filename: Response_to_Reviewers_auresp_4.pdf [file pone.0351879.s007.pdf]

Response to Reviewers' Comments

Manuscript Title: Case-control study of risk factors for abscess development following hepatitis B vaccination in children in Timor-Leste, 2024

Manuscript ID: PONE-D-26-02442R3

Dear Editor and Reviewers,

Thank you for your thoughtful and constructive review of our manuscript. We appreciate your recognition of the substantive improvements made in response to previous revisions and your clear guidance on the remaining minor issues. We have addressed each of the three items as requested.

Below, we respond point-by-point to your comments.

Comment 1: Inconsistent footnote duplication in Table 4

Editor's comment:

"In the revised manuscript (pages 27-28 of the PDF, and again on pages 57-58), Table 4 contains two identical footnotes labeled with the same asterisk symbol... This duplication is redundant and should be consolidated into a single footnote placed directly beneath the table.

Required action: Keep only one version of the bolded instability footnote. Remove the duplicate. Retain the separate footnote about cold chain variables (marked with a different symbol, e.g., † or a)."

Authors' response:
We have consolidated the duplicate footnotes into a single bolded instability statement placed directly beneath Table 4. The cold chain variables footnote now uses a distinct symbol (†) to avoid confusion. The revised Table 4 footnote now reads:

*Due to the very small number of discordant pairs (≤4 for all variables), matched odds ratios, confidence intervals, and p-values are statistically unstable and should not be interpreted as reliable measures of effect magnitude or evidence for or against association.

†Cold chain variables (temperature monitoring and physical storage conditions) were excluded from matched analysis because no discordant pairs were observed across matched case–control pairs.

Action taken: Completed. The duplicate footnote has been removed.

Comment 2: Minor inconsistency in the Discussion

Editor's comment:

"Page 29 (and page 59) states: 'The discordant pair pattern for multidose vial status (4 vs. 1) is compatible with a very wide range of possible effects, from no association to a substantial increase in risk.' This is acceptable and appropriately neutral. However, the very next sentence says: 'No meaningful inference can be drawn from this pattern alone.' The word 'alone' is unnecessary because the preceding sentence already establishes that no precise inference is possible. Delete 'alone' to avoid any residual suggestion that additional information might salvage the inference."

Authors' response:
We have deleted the word "alone" from the sentence as requested. The revised sentence now reads:

"No meaningful inference can be drawn from this pattern."

Action taken: Completed. The word "alone" has been removed from the Discussion section.

Comment 3: Abstract – redundant phrasing

Editor's comment:

"The abstract's Results section (page 13, line 39-44) reads: 'None of the examined variables showed statistically significant associations… and confidence intervals were extremely wide, reflecting the small sample size (12 pairs).' The subsequent sentence in the abstract says: 'The very small number of discordant pairs (≤4 for all comparisons) resulted in substantial statistical instability, precluding any reliable estimation of associations.' This is repetitive. Combine or remove the second sentence to avoid verbatim overlap. I suggest keeping only: 'None of the examined variables showed statistically significant associations; confidence intervals were extremely wide, reflecting the small sample size (12 pairs) and the very small number of discordant pairs (≤4 for all comparisons).'"

Authors' response:
We have streamlined the abstract's Results section into a single sentence as suggested. The revised text now reads:

"None of the examined variables showed statistically significant associations with injection-site abscess formation; confidence intervals were extremely wide, reflecting the small sample size (12 pairs) and the very small number of discordant pairs (≤4 for all comparisons). Findings should therefore be interpreted as descriptive and hypothesis-generating only.."

Action taken: Completed. The repetitive phrasing has been removed.

Summary of Changes

Editor Comment	Action Taken
Duplicate footnote in Table 4	Consolidated into a single bolded instability footnote; retained a separate footnote for cold chain variables using † symbol
Unnecessary word "alone" in Discussion	Removed "alone" from the sentence
Repetitive phrasing in Abstract Results	Streamlined into a single combined sentence

Declaration

We confirm that:

1. No other changes have been made to the manuscript beyond the three specific corrections requested above.
2. The scientific content, statistical interpretations, and overall framing remain exactly as approved in principle by the Editor.
3. The manuscript is not under consideration elsewhere and has been approved by all authors.

We believe the manuscript now meets PLOS ONE's publication criteria and respectfully submit this corrected version for final acceptance.

Thank you again for your fair and constructive review, and for your recognition of our efforts to present this small study with appropriate transparency and scientific honesty.

Attachments:

Revised manuscript with track changes

Clean revised manuscript

Corresponding author:
Mariano da Silva Marques
Instituto Nacional da Saúde Pública Timor-Leste
Email: dr.marquess31@gmail.com
