# Supplementary material for: Case-control study of risk factors for abscess development following hepatitis B vaccination in children in Timor-Leste, 2024
Source: PLoS One. 2026 Jun 22;21(6):e0351879. doi: 10.1371/journal.pone.0351879 (PMC13286217; doi:10.1371/journal.pone.0351879)
Supplement: Peer review history

# Peer review: Decision Letter 0

**DOI:** 10.1371/journal.pone.0351879.r001

**Authors:** Le An Pham

11 Mar 2026

PONE-D-26-02442

Case control study of risk factors for abscess development following hepatitis B vaccination in children in Timor-Leste, 2024

PLOS One

Dear Dr. da Silva Marques,

Thank you for submitting your manuscript to PLOS ONE. After careful consideration, we feel that it has merit but does not fully meet PLOS ONE’s publication criteria as it currently stands. Therefore, we invite you to submit a revised version of the manuscript that addresses the points raised during the review process.

Please submit your revised manuscript by Apr 25 2026 11:59PM. If you will need more time than this to complete your revisions, please reply to this message or contact the journal office at plosone@plos.org. When you're ready to submit your revision, log on to https://www.editorialmanager.com/pone/ and select the ’submissions Needing Revision' folder to locate your manuscript file.

Please include the following items when submitting your revised manuscript:

- A letter that responds to each point raised by the academic editor and reviewer(s). You should upload this letter as a separate file labeled 'Response to Reviewers'.
- A marked-up copy of your manuscript that highlights changes made to the original version. You should upload this as a separate file labeled 'Revised Manuscript with Track Changes'.
- An unmarked version of your revised paper without tracked changes. You should upload this as a separate file labeled 'Manuscript'.

If you would like to make changes to your financial disclosure, please include your updated statement in your cover letter. Guidelines for resubmitting your figure files are available below the reviewer comments at the end of this letter.

If applicable, we recommend that you deposit your laboratory protocols in protocols.io to enhance the reproducibility of your results. Protocols.io assigns your protocol its own identifier (DOI) so that it can be cited independently in the future. For instructions see: https://journals.plos.org/plosone/s/submission-guidelines#loc-laboratory-protocols. Additionally, PLOS ONE offers an option for publishing peer-reviewed Lab Protocol articles, which describe protocols hosted on protocols.io. Read more information on sharing protocols at https://plos.org/protocols?utm_medium=editorial-email&utm_source=authorletters&utm_campaign=protocols.

We look forward to receiving your revised manuscript.

Kind regards,

Le An Pham, Ph.D.,M.D.

Academic Editor

PLOS One

Journal Requirements:

When submitting your revision, we need you to address these additional requirements.

1. Please ensure that your manuscript meets PLOS ONE’s style requirements, including those for file naming. The PLOS ONE style templates can be found at

https://journals.plos.org/plosone/s/file?id=wjVg/PLOSOne_formatting_sample_main_body.pdf and

https://journals.plos.org/plosone/s/file?id=ba62/PLOSOne_formatting_sample_title_authors_affiliations.pdf

2. We note that your Data Availability Statement is currently as follows: “All relevant data are within the manuscript and its Supporting Information files.”

Please confirm at this time whether or not your submission contains all raw data required to replicate the results of your study. Authors must share the “minimal data set” for their submission. PLOS defines the minimal data set to consist of the data required to replicate all study findings reported in the article, as well as related metadata and methods (https://journals.plos.org/plosone/s/data-availability#loc-minimal-data-set-definition).

For example, authors should submit the following data:

- The values behind the means, standard deviations and other measures reported;

- The values used to build graphs;

- The points extracted from images for analysis.

Authors do not need to submit their entire data set if only a portion of the data was used in the reported study.

If your submission does not contain these data, please either upload them as Supporting Information files or deposit them to a stable, public repository and provide us with the relevant URLs, DOIs, or accession numbers. For a list of recommended repositories, please see https://journals.plos.org/plosone/s/recommended-repositories.

If there are ethical or legal restrictions on sharing a de-identified data set, please explain them in detail (e.g., data contain potentially sensitive information, data are owned by a third-party organization, etc.) and who has imposed them (e.g., an ethics committee). Please also provide contact information for a data access committee, ethics committee, or other institutional body to which data requests may be sent. If data are owned by a third party, please indicate how others may request data access.

3. Please amend either the title on the online submission form (via Edit Submission) or the title in the manuscript so that they are identical.

4. Please amend the manuscript submission data (via Edit Submission) to include author Marcelo Amaral Mali, Nevio da Costa Sarmento, Domingos Soares, Noel Gama Soares, Cristovão de Alexandria Barros, Livia Natalia Maria Guterres Babo, Armandinha A. da C. Silva, Evaliza Adão Mendonça, Natalia Jesus da Silva, Nazario Barreto dos Santos and Marck A. A. Magno Neves.

5. If the reviewer comments include a recommendation to cite specific previously published works, please review and evaluate these publications to determine whether they are relevant and should be cited. There is no requirement to cite these works unless the editor has indicated otherwise.

Additional Editor Comments:

The study includes only 12 cases and 12 controls, and in the final multivariable model no factor remained statistically significant; the confidence intervals are extremely wide, showing major instability. Yet the abstract and conclusion still state that omission of skin antisepsis was the “critical” or “primary” modifiable risk factor

A second major problem is internal inconsistency in the methods/statistics. The manuscript says cases and controls were matched by age, vaccination date, and clinic, but the statistical analysis section says the conditional logistic models were conditioned on age, sex, and vaccination site.

There are also important bias and measurement limitations. Exposure data were collected retrospectively in late 2025 about vaccination events from 2024, including caregiver interviews and vaccinator recall; the manuscript itself acknowledges recall and social-desirability bias

[Note: HTML markup is below. Please do not edit.]

Reviewers' comments:

Reviewer’s Responses to Questions

**Comments to the Author**

1. Is the manuscript technically sound, and do the data support the conclusions?

The manuscript must describe a technically sound piece of scientific research with data that supports the conclusions. Experiments must have been conducted rigorously, with appropriate controls, replication, and sample sizes. The conclusions must be drawn appropriately based on the data presented.

Reviewer #1: Partly

Reviewer #2: No

Reviewer #3: No

Reviewer #4: No

**********

2. Has the statistical analysis been performed appropriately and rigorously?

Reviewer #1: N/A

Reviewer #2: No

Reviewer #3: No

Reviewer #4: No

**********

3. Have the authors made all data underlying the findings in their manuscript fully available?

The PLOS Data policy requires authors to make all data underlying the findings described in their manuscript fully available without restriction, with rare exception (please refer to the Data Availability Statement in the manuscript PDF file). The data should be provided as part of the manuscript or its supporting information, or deposited to a public repository. For example, in addition to summary statistics, the data points behind means, medians and variance measures should be available. If there are restrictions on publicly sharing data—e.g. participant privacy or use of data from a third party—those must be specified.

Reviewer #1: Yes

Reviewer #2: No

Reviewer #3: Yes

Reviewer #4: Yes

**********

4. Is the manuscript presented in an intelligible fashion and written in standard English?

PLOS ONE does not copyedit accepted manuscripts, so the language in submitted articles must be clear, correct, and unambiguous. Any typographical or grammatical errors should be corrected at revision, so please note any specific errors here.

Reviewer #1: No

Reviewer #2: Yes

Reviewer #3: Yes

Reviewer #4: No

**********

5. Review Comments to the Author

Please use the space provided to explain your answers to the questions above. You may also include additional comments for the author, including concerns about dual publication, research ethics, or publication ethics. (Please upload your review as an attachment if it exceeds 20,000 characters)

Reviewer #1: General Comments

The manuscript addresses an important issue and has strong public health relevance. However, significant methodological clarifications and revisions are required, particularly regarding statistical interpretation, internal consistency of the results, and cautious interpretation of the findings. I recommend major revision before the manuscript can be considered for publication.

Major Comments

The authors are kindly requested to:

- Update the background information regarding serious adverse events following immunization (AEFIs) after hepatitis B (HBV) vaccination, including current global estimates and references.

- Add a detailed description of how the sample size was calculated.

- Clarify how study controls were selected.

- Provide clearer definitions of key variables. For example: Regular monitoring of vaccine storage temperature, physical conditions of vaccine storage, whether the administered dose was taken from a newly opened multi-dose vial (as presented in Table 3).

- The criterion “aged < 12 months at the time of vaccination in 2024” appears to be an inclusion criterion rather than a study variable, as presented in Table 1, and should be revised accordingly.

- Gestational age is categorized as < 37 weeks and ≥ 37 weeks; however, Table 2 presents categories as < 36 and ≥ 36 weeks. The authors need to ensure consistency throughout the manuscript and provide references supporting the selected cut-off definition

- The study includes only 12 cases and 12 matched controls. This extremely small sample size severely limits statistical power and results in very wide confidence intervals in both bivariate and multivariable analyses (e.g., aOR = 23.19; 95% CI: 0.15–3605.50), which preclude definitive conclusions for some associations.

- Clarify whether all eligible cases in 2024 were included. Lines 96–100 report that:

“According to the national surveillance data for 2024, 62 cases of AEFI were reported. Within this cohort, 89% of cases involved abscess formation, with the affected demographic predominantly comprising children under the age of one year. These events occurred during routine immunization. Of the total reported AEFI cases, 95% were classified as serious.”

This statement requires clarification regarding how the final 12 cases were selected from the reported cases.

- In Table 3, antiseptic omission is reported as 91.7% in both cases and controls. However, a strong association (aOR = 11.0) is reported. If exposure prevalence was identical in both groups, the odds ratio should not indicate a strong association. This discrepancy requires clarification

- Cold chain variables show 100% proper practice in both groups, yet odds ratios are reported (e.g., OR = 0.04 with CI). Statistically, when there is no variation in exposure, meaningful estimation is not possible. The same concern applies to the “work experience of vaccinator” variable if no variability exists

- Exposure data were partially collected through retrospective caregiver interviews and healthcare worker recall of practices in 2024. This introduces a substantial risk of: recall bias, social desirability bias, misclassification bias. The limitations section should more explicitly acknowledge how these biases may have influenced the findings

Minor Comments

- Grammar and language require careful revision (e.g., “left thigh” is sometimes written as “left tight”).

- Some sentences are overly long and could be simplified to improve clarity.

Reviewer #2: This manuscript examines risk factors associated with injection-site abscesses following hepatitis B vaccination in infants using a matched case-control design based on national AEFI surveillance data in Timor-Leste. The topic is important and highly relevant to vaccine safety, particularly in low-resource settings where immunization program quality and public confidence in vaccination programs are critical. However, in its current form the study has several methodological and statistical limitations that substantially restrict the strength and reliability of the conclusions.

1. Very small sample size and unreliable statistical modeling

The study includes only 12 cases and 12 matched controls, which severely limits statistical power and the reliability of estimated associations. This limitation is compounded by the use of a multivariable conditional logistic regression model including multiple predictors. With such a small number of outcome events, the model is likely to be severely overfitted, resulting in unstable estimates and extremely wide confidence intervals (e.g., aOR 23.19; 95% CI 0.15–3605.50). Under these conditions, the regression analysis cannot provide reliable estimates of independent risk factors. Consequently, the analytical approach does not adequately support the conclusions drawn in the manuscript.

2. Inconsistency in the antiseptic exposure variable

The manuscript highlights omission of skin antisepsis as a key risk factor. However, the data presented in Table 3 indicate that antiseptic use appears nearly identical between cases and controls. This apparent discrepancy between the exposure distribution and the reported odds ratio requires clarification. The authors should carefully review the statistical calculations and ensure that the analytical methods used for matched data have been correctly applied. Because this variable forms the central argument of the manuscript, this issue is particularly important.

3. Potential recall bias in exposure data

Exposure data were collected retrospectively through interviews conducted approximately one year after the vaccination events. Procedural details such as antiseptic use or vaccine vial handling may be difficult for vaccinators or caregivers to recall accurately after such a long interval. This introduces substantial potential for recall bias and exposure misclassification, which could influence the observed associations.

4. Outcome definition and diagnostic criteria

The study defines cases as clinically confirmed abscesses within 14 days of vaccination, but the diagnostic criteria used to confirm these cases are not fully described. Without standardized diagnostic criteria or microbiological confirmation, some reported cases could represent sterile abscesses or inflammatory injection-site reactions rather than true infectious abscesses.

5. Minor comments

- Correct typographical errors such as “left tight”, which likely should be “left thigh.”

- Clarify matching variables and ensure consistency between the study design description and the statistical analysis section.

- Avoid presenting odds ratios in purely descriptive tables.

- Cold chain variables show no variation between groups and therefore cannot be meaningfully analyzed.

Due to the very limited sample size, concerns regarding the statistical analysis, and potential exposure measurement bias, the current analysis does not provide sufficiently robust evidence to support the conclusions presented. For these reasons, I recommend that the manuscript not be accepted in its current form.

Reviewer #3: The manuscript is written in clear, standard English; however, several minor formatting and proofreading issues should be addressed, such as typographical errors in Table 2 and Table 5 (e.g., “left tight” and “right tight”). In addition, the titles of the tables and figures should be renamed for consistency and clarity.

More importantly, the manuscript contains several critical mathematical impossibilities and methodological inconsistencies:

• An impossible adjusted odds ratio (aOR) of 11.0 is reported for identical exposures in Table 3.

• Despite a 0% variation between the two groups (no instances of improper practice in either group), the authors report an aOR of 0.04 with a statistically significant p-value in Table 4.

• An aOR of 0.04 is presented for gestational age in Table 2, reflecting an incorrect interpretation and directionality of the odds ratios. Since the case group had a higher rate of preterm birth, this variable should theoretically function as a risk factor (OR > 1).

• The study relies on a tiny sample size of only 24 participants, raising concerns about the validity and stability of the statistical estimates.

Overall, the manuscript is technically unsound, and its conclusions are unsupported, as the primary findings depend on statistically impossible outputs that directly contradict the raw descriptive data. A full re-evaluation of the statistical methods and results is required.

Reviewer #4: Summary

This manuscript investigates risk factors associated with injection-site abscess formation following hepatitis B vaccination in infants in Timor-Leste using a retrospective matched case–control design. The topic is important for vaccine safety and immunization program quality in low-resource settings. However, several methodological limitations, particularly the very small sample size and inconsistencies in the analysis, limit the strength of the conclusions.

Major comments:

1. Very small sample size

The study includes only 12 cases and 12 controls. This severely limits statistical power and leads to extremely wide confidence intervals in the regression models. The findings should therefore be interpreted cautiously and framed as exploratory rather than definitive. For example, the author concludes that “The near-universal omission of skin disinfection was identified as a critical, modifiable risk factor for post-vaccination abscesses in this setting” or “Results from this study show the critical importance of strict adherence to aseptic technique, specifically skin disinfection, as the most immediate strategy to prevent injection-site abscesses.” Or “This study identifies a significant breach in aseptic technique, specifically the omission of skin antisepsis, as the primary modifiable risk factor for post-vaccination abscesses.”. The author should revise the word use.

2. Inconsistency in antiseptic use results

The manuscript reports that antiseptic use was omitted in 91.7% of both cases and controls, yet a strong association (aOR = 11.0) is reported (Table 3). The authors should clarify how this estimate was derived from matched analysis, as identical exposure distributions between groups would normally not produce such an association.

3. Potential recall bias

Several key exposure variables (e.g., antiseptic use and vaccination practices) were assessed retrospectively through caregiver and vaccinator recall for events occurring the previous year. This introduces potential recall and reporting bias, which should be more clearly acknowledged in the discussion.

4. Overfitting in multivariable regression

Given the small number of case–control pairs, the multivariable regression model likely suffers from overfitting, so may not be suitable. The authors should consider simplifying the analysis or presenting mainly the matched bivariate results.

Minor comments

• Several language and typographical errors should be corrected (e.g., “Left tight” should be “Left thigh,” and “multivial dose” should be “multidose vial”).

• Table titles should be simplified and redundant wording removed.

• The clinical definition of abscess should be clarified.

• The spacing between rows in the tables should be reduced.

• Figure 1 is unclear.

Recommendation

The study addresses an important operational issue in immunization safety, but methodological clarification and more cautious interpretation of results are required before the manuscript can be considered for publication.

**********

6. PLOS authors have the option to publish the peer review history of their article (what does this mean?). If published, this will include your full peer review and any attached files.

If you choose “no”, your identity will remain anonymous but your review may still be made public.

**Do you want your identity to be public for this peer review?**   For information about this choice, including consent withdrawal, please see our Privacy Policy.

Reviewer #1: No

Reviewer #2: No

Reviewer #3: No

Reviewer #4: No

**********

[NOTE: If reviewer comments were submitted as an attachment file, they will be attached to this email and accessible via the submission site. Please log into your account, locate the manuscript record, and check for the action link "View Attachments". If this link does not appear, there are no attachment files.]

To ensure your figures meet our technical requirements, please review our figure guidelines: https://journals.plos.org/plosone/s/figures

You may also use PLOS’s free figure tool, NAAS, to help you prepare publication quality figures: https://journals.plos.org/plosone/s/figures#loc-tools-for-figure-preparation.

NAAS will assess whether your figures meet our technical requirements by comparing each figure against our figure specifications.

# Author comment: Author response to Decision Letter 1

**DOI:** 10.1371/journal.pone.0351879.r002

16 Mar 2026

Response to Reviewers' Comments

Manuscript Title: Case-control study of risk factors for abscess development following hepatitis B vaccination in children in Timor-Leste, 2024

Dear Editor and Reviewers,

We sincerely thank the reviewers for their careful evaluation of our manuscript and for their constructive comments. The suggestions have been extremely valuable in improving the clarity, methodological transparency, and statistical interpretation of the study.

We have revised the manuscript accordingly. Major revisions include clarification of case selection procedures, revision of statistical analyses for matched data, removal of variables with no variability across matched pairs, cautious interpretation of results given the small sample size, and an expanded discussion of potential biases and limitations.

All changes have been incorporated in the revised manuscript. Detailed responses to each reviewer comment are provided below.

RESPONSE TO REVIEWER #1

General Comment: The manuscript addresses an important issue and has strong public health relevance. However, significant methodological clarifications and revisions are required, particularly regarding statistical interpretation, internal consistency of the results, and cautious interpretation of the findings. I recommend major revision before the manuscript can be considered for publication.

Response: We thank the reviewer for their thoughtful and constructive comments, which have substantially improved the manuscript. We have carefully addressed each point raised and provide a point-by-point response below. All changes have been marked in the revised manuscript using track changes.

Major Comments

a) Update the background information regarding serious adverse events following immunization (AEFIs) after hepatitis B (HBV) vaccination, including current global estimates and references.

Response: Thank you for this valuable suggestion. We have updated the background section to include more current global estimates and recent references on serious AEFIs following hepatitis B vaccination. Specifically, we have:

• Added the most recent WHO position paper on hepatitis B vaccines (2019, referenced as [8])

• Included updated pharmacovigilance data from the US Vaccine Adverse Event Reporting System (VAERS) through 2023 (referenced as [16])

• Added regional data from the WHO Southeast Asia Region on injection-site abscesses associated with hepatitis B vaccination (referenced as [17])

The revised text now reads (lines 91-103):

"The hepatitis B vaccine is widely recognized for its safety [8], being associated primarily with mild and self-limited adverse events [9]. The most common adverse reactions to the hepatitis B vaccine are local and transient systemic symptoms, typically occurring within 24 hours of administration. These include pain at the injection site (with an incidence of 3--29 per 100 doses), erythema (3 per 100), and swelling (3 per 100). Mild systemic symptoms such as fever (1--6 per 100) and headache (3 per 100) have also been documented[9-11]. Severe reactions such as anaphylaxis occur at an estimated rate of approximately 1--2 cases per million vaccine doses administered [9, 12]. Notably, declines in hepatitis B vaccination coverage have been observed following media reports of infant deaths after administration of the hepatitis B vaccine birth dose (HepB-BD) in Vietnam [13] and China [14, 15]. As post-marketing data accumulate, several serious adverse events following hepatitis B vaccination have been reported. The most recent pharmacovigilance analysis in the United States suggests that adverse events associated with the hepatitis B vaccine include general disorders and administration site conditions, nervous system disorders, aplastic anaemia, exfoliative dermatitis, and haemolytic anaemia [16]. However, quantitative studies based on real-world data remain limited, and comprehensive information on these adverse events is still lacking[16]."

b) Add a detailed description of how the sample size was calculated.

Response: We acknowledge that the original manuscript did not include a sample size calculation. This was an exploratory study conducted as part of the national AEFI outbreak response, and we included all eligible cases identified through the national surveillance system during the study period, rather than recruiting based on a pre-specified sample size calculation. We have now clarified this in the Methods section and added text explaining that the study included all available cases meeting eligibility criteria.

The revised Methods section now states (lines 176-187):

"Cases were eligible for inclusion if they met the following criteria:

• Age <12 months at the time of vaccination

• Receipt of hepatitis B birth-dose vaccine during routine immunization services in 2024

• Development of a clinically confirmed injection-site abscess within 14 days following vaccination

• Availability of vaccination records and caregiver contact for interview

Cases were excluded if clinical documentation was incomplete, if the diagnosis could not be verified, or if caregivers could not be contacted for follow-up interviews.

After applying eligibility criteria and matching requirements, 12 cases with complete data and successfully matched controls were included in the final analysis (Fig 2)."

c) Clarify how study controls were selected.

Response: Thank you for requesting this clarification. We have expanded the description of control selection in the Methods section to provide greater detail on the sampling strategy.

The revised text now reads (lines 196-212):

"Controls were infants who received the hepatitis B birth-dose vaccine during the same time period and at the same health facility as the corresponding case but did not develop any reported adverse event following immunization during the observation period.

For each eligible case, one control was selected from the same health facility vaccination register. Controls were infants who received the hepatitis B birth-dose vaccine during the same period but did not develop any reported AEFI during the 14-day observation period.

Controls were matched to cases based on:

• Vaccination facility

• Approximate vaccination date (±7 days)

• Infant age group

• Sex

This matching strategy was intended to control for facility-level practices, vaccine storage conditions, and temporal variation in immunization services. All controls were confirmed through review of vaccination records and caregiver interview to have no documented or reported AEFI within 14 days following vaccination."

d) Provide clearer definitions of key variables. For example: Regular monitoring of vaccine storage temperature, physical conditions of vaccine storage, whether the administered dose was taken from a newly opened multi-dose vial (as presented in Table 3).

Response: We agree that clearer operational definitions are essential. We have revised Table 1 to provide more detailed and precise definitions for all key variables, including those specifically mentioned. The revised Table 1 (lines 257-302) now includes detailed operational definitions and measurement classifications for variables such as Temperature Monitoring, Vaccine Storage Conditions, and Multidose Vial Policy.

e) The criterion "aged < 12 months at the time of vaccination in 2024" appears to be an inclusion criterion rather than a study variable, as presented in Table 1, and should be revised accordingly.

Response: Thank you for identifying this error. You are correct that age <12 months was an inclusion criterion, not an exposure variable. We have removed this from Table 1 and have clarified in the inclusion criteria section (lines 176-179) that this was an eligibility requirement. The variable "Infant age group" that was used for matching is now described in the matching criteria section (line 208) rather than as an exposure variable.

f) Gestational age is categorized as < 37 weeks and ≥ 37 weeks; however, Table 2 presents categories as < 36 and ≥ 36 weeks. The authors need to ensure consistency throughout the manuscript and provide references supporting the selected cut-off definition.

Response: We thank the reviewer for identifying this inconsistency. This was a typographical error in Table 2. We have corrected Table 2 (lines 333-359) to consistently show "<37 weeks" and "≥37 weeks" throughout the manuscript. We have also added a reference supporting the use of 37 weeks as the standard cut-off for preterm birth, consistent with WHO definitions in Table 1 (line 295) and the References section [31].

g) The study includes only 12 cases and 12 matched controls. This extremely small sample size severely limits statistical power and results in very wide confidence intervals in both bivariate and multivariable analyses (e.g., aOR = 23.19; 95% CI: 0.15--3605.50), which preclude definitive conclusions for some associations.

Response: We fully acknowledge this critical limitation. Throughout the revised manuscript, we have substantially tempered our language and emphasized the exploratory nature of the findings. We have:

1. Added explicit statements about the small sample size as a major limitation in the Abstract, Methods, Results, and Discussion sections

2. Removed all definitive language suggesting causal associations

3. Framed the study as hypothesis-generating rather than hypothesis-testing

4. Emphasized that the wide confidence intervals reflect statistical uncertainty and preclude definitive conclusions

For example, the Abstract conclusions now read (lines 56-63):

"In this exploratory matched case--control study, no statistically significant independent risk factors for injection-site abscess following hepatitis B birth-dose vaccination were identified. The findings should be interpreted cautiously because of the small sample size and limited statistical power. Strengthening safe injection practices, appropriate multidose vial management, and AEFI surveillance systems remains important to support vaccine safety and maintain public confidence in immunization programs. Larger studies are needed to further investigate potential determinants of abscess formation."

h) Clarify whether all eligible cases in 2024 were included. Lines 96--100 report that: "According to the national surveillance data for 2024, 62 cases of AEFI were reported. Within this cohort, 89% of cases involved abscess formation, with the affected demographic predominantly comprising children under the age of one year. These events occurred during routine immunization. Of the total reported AEFI cases, 95% were classified as serious." This statement requires clarification regarding how the final 12 cases were selected from the reported cases.

Response: Thank you for requesting this clarification. We have added a detailed case selection flow diagram (Figure 2, line 189) and explanatory text describing the stepwise selection process. The revised text now reads (lines 176-187, as detailed in response b).

i) In Table 3, antiseptic omission is reported as 91.7% in both cases and controls. However, a strong association (aOR = 11.0) is reported. If exposure prevalence was identical in both groups, the odds ratio should not indicate a strong association. This discrepancy requires clarification.

Response: We thank the reviewer for identifying this critical error. This was a major oversight in our original manuscript. Upon re-examination of the data and statistical code, we discovered that:

1. The exposure prevalence was indeed nearly identical between groups (91.7% vs 91.7%), which mathematically cannot produce an odds ratio substantially different from 1.0 in a properly specified model.

2. The reported aOR of 11.0 was a transcription error from an earlier exploratory model that included interaction terms and had been incorrectly specified.

3. In the corrected analysis using the exact McNemar test for matched pairs, the matched odds ratio for antiseptic application is 1.00 (95% CI: 0.06-18.09), with a p-value of 0.006 from the exact McNemar test (reflecting the small number of discordant pairs).

We have:

• Corrected this error throughout the manuscript

• Removed all references to antiseptic omission as a "critical risk factor"

• Added explanatory text about why the McNemar test p-value may be significant even with mOR=1.00 when based on very sparse data

• Excluded this variable from multivariable modeling due to instability

The revised Table 5 (lines 420-443) now correctly shows:

Variable Comparison Matched Odds Ratio (mOR) 95% CI p-value

Antiseptic application Yes vs No 1.00 0.06-18.09 0.006

We have added the following explanatory note in the results section (lines 457-464):

"Application of an antiseptic at the injection site prior to vaccination showed a statistically significant difference between cases and controls based on the exact McNemar test (p = 0.006). However, the matched odds ratio was 1.00 with a wide confidence interval (95% CI: 0.06--18.09), indicating instability of the estimate due to the small number of discordant pairs. Therefore, this finding should be interpreted cautiously."

And further in the discussion (lines 538-546):

"The statistically significant p-value observed for antiseptic application in the McNemar analysis should be interpreted cautiously. The matched odds ratio was unstable and associated with a very wide confidence interval, reflecting the extremely small number of discordant pairs. For this reason, the variable was excluded from the multivariable regression model to avoid model instability. Importantly, this analytical limitation should not be interpreted as evidence that skin antisepsis is unimportant, as appropriate skin preparation remains a key component of safe injection practice and infection prevention [22]."

j) Cold chain variables show 100% proper practice in both groups, yet odds ratios are reported (e.g., OR = 0.04 with CI). Statistically, when there is no variation in exposure, meaningful estimation is not possible. The same concern applies to the "work experience of vaccinator" variable if no variability exists.

Response: Thank you for identifying this statistical impossibility. These were errors in our original table presentation. We have:

1. Removed all odds ratios and confidence intervals for variables with zero variability (cold chain variables)

2. Clearly indicated in all tables and text that these variables could not be analyzed due to absence of variation

3. Noted in the limitations section that the uniform documentation of proper cold chain practices may reflect either true compliance or limitations in routine documentation to detect transient deviations

The revised Table 4 (lines 393-408) now simply presents the descriptive frequencies without any measures of association, with a footnote (implied by the text) that due to zero variability, measures of association could not be estimated. The text above the table states: "Vaccine storage and handling practices were uniformly proper for all participants (Table 4)."

k) Exposure data were partially collected through retrospective caregiver interviews and healthcare worker recall of practices in 2024. This introduces a substantial risk of: recall bias, social desirability bias, misclassification bias. The limitations section should more explicitly acknowledge how these biases may have influenced the findings.

Response: We agree and have substantially expanded the limitations section to explicitly address these biases. The revised text now reads (lines 576-596):

"Second, the retrospective design introduces the possibility of recall and information bias, particularly for provider practice variables such as antiseptic use and multidose vial handling. These exposures were assessed approximately one year after the vaccination events and may therefore have been subject to inaccurate recall or social desirability bias."

We have elaborated further earlier in the limitations section to explicitly address these biases (lines 580-596):

"Second, the retrospective design introduces substantial potential for information bias. Key exposure variables---including antiseptic use, multidose vial handling practices, and details of the vaccination procedure---were assessed through interviews conducted approximately one year after the v

Attachment Submitted filename: Response to Reviewer.pdf

# Peer review: Decision Letter 1

**DOI:** 10.1371/journal.pone.0351879.r003

**Authors:** Le An Pham

24 Mar 2026

<div>PONE-D-26-02442R1

Case control study of risk factors for abscess development following hepatitis B vaccination in children in Timor-Leste, 2024

PLOS One

Dear Dr. da Silva Marques,

Thank you for submitting your manuscript to PLOS ONE. After careful consideration, we feel that it has merit but does not fully meet PLOS ONE’s publication criteria as it currently stands. Therefore, we invite you to submit a revised version of the manuscript that addresses the points raised during the review process.

My recommendation is **one more revision**  .

1. **Recheck the antiseptic analysis from the raw matched-pair counts.**
  This is the biggest issue. The authors should present the discordant pair counts explicitly and make sure the matched odds ratio, confidence interval, and McNemar p-value all come from the same data and method. If the mOR is truly 1.00, the p-value likely needs correction. If the p-value is truly 0.006, the mOR or its rounding/reporting likely needs correction.
2. **Consider removing the conditional logistic regression entirely.**
  With only 12 matched pairs, even a 3-variable model is still fragile. The paper would probably be stronger if it relied mainly on descriptive matched analysis and stated clearly that the study was underpowered for robust multivariable inference. The authors already moved in this direction, and going further would improve credibility.
3. **Reframe the study aim slightly.**
  Instead of “identify risk factors,” use language like:
  “explore potential factors associated with abscess occurrence during an AEFI investigation.”
  That wording better matches what the data can support.
4. **Add a clearer selection flow from the surveillance pool to the final 12 pairs.**
  The reviewers asked about how the final 12 were derived from the larger 2024 AEFI reports. The authors say they added a flow diagram, which is good, but this needs to be very explicit and easy to follow in the manuscript.
5. **State very clearly that absence of significant association does not mean absence of risk.**
  The revised paper is better on this point already, but it should say plainly that the study may have missed true associations because of low power, misclassification, and recall bias.
6. **Move zero-variation variables to descriptive text or supplement.**
  Since cold-chain variables could not be analytically estimated, keeping them minimal will tighten the paper.

Please submit your revised manuscript by May 08 2026 11:59PM. If you will need more time than this to complete your revisions, please reply to this message or contact the journal office at plosone@plos.org. When you're ready to submit your revision, log on to https://www.editorialmanager.com/pone/ and select the ’submissions Needing Revision' folder to locate your manuscript file.

Please include the following items when submitting your revised manuscript:

- A letter that responds to each point raised by the academic editor and reviewer(s). You should upload this letter as a separate file labeled 'Response to Reviewers'.
- A marked-up copy of your manuscript that highlights changes made to the original version. You should upload this as a separate file labeled 'Revised Manuscript with Track Changes'.
- An unmarked version of your revised paper without tracked changes. You should upload this as a separate file labeled 'Manuscript'.

If you would like to make changes to your financial disclosure, please include your updated statement in your cover letter. Guidelines for resubmitting your figure files are available below the reviewer comments at the end of this letter.

If applicable, we recommend that you deposit your laboratory protocols in protocols.io to enhance the reproducibility of your results. Protocols.io assigns your protocol its own identifier (DOI) so that it can be cited independently in the future. For instructions see: https://journals.plos.org/plosone/s/submission-guidelines#loc-laboratory-protocols. Additionally, PLOS ONE offers an option for publishing peer-reviewed Lab Protocol articles, which describe protocols hosted on protocols.io. Read more information on sharing protocols at https://plos.org/protocols?utm_medium=editorial-email&utm_source=authorletters&utm_campaign=protocols.

As the corresponding author, your ORCID iD is verified in the submission system and will appear in the published article. PLOS supports the use of ORCID, and we encourage all coauthors to register for an ORCID iD and use it as well. Please encourage your coauthors to verify their ORCID iD within the submission system before final acceptance, as unverified ORCID iDs will not appear in the published article. *Only*   the individual author can complete the verification step; PLOS staff *cannot*   verify ORCID iDs on behalf of authors.

We look forward to receiving your revised manuscript.

Kind regards,

Le An Pham, Ph.D.,M.D.

Academic Editor

PLOS One

Journal Requirements:

If the reviewer comments include a recommendation to cite specific previously published works, please review and evaluate these publications to determine whether they are relevant and should be cited. There is no requirement to cite these works unless the editor has indicated otherwise.

Additional Editor Comments:

The revised version is substantially better than the original in almost every important way:

better methods description,

better alignment of conclusions with evidence,

better limitations,

less overclaiming,

cleaner tables and presentation.

But I would still call it not fully safe yet, because the antiseptic result appears to remain internally inconsistent. So my final judgment is:

Strength: strong improvement, much more publishable than the original.

Weakness: one key statistical issue still looks unresolved, and the study remains very underpowered.

Best adjustment: do one more statistical cleanup and make the paper purely exploratory.

My recommendation is one more revision.

Recheck the antiseptic analysis from the raw matched-pair counts.

This is the biggest issue. The authors should present the discordant pair counts explicitly and make sure the matched odds ratio, confidence interval, and McNemar p-value all come from the same data and method. If the mOR is truly 1.00, the p-value likely needs correction. If the p-value is truly 0.006, the mOR or its rounding/reporting likely needs correction.

Consider removing the conditional logistic regression entirely.

With only 12 matched pairs, even a 3-variable model is still fragile. The paper would probably be stronger if it relied mainly on descriptive matched analysis and stated clearly that the study was underpowered for robust multivariable inference. The authors already moved in this direction, and going further would improve credibility.

Reframe the study aim slightly.

Instead of “identify risk factors,” use language like:

“explore potential factors associated with abscess occurrence during an AEFI investigation.”

That wording better matches what the data can support.

Add a clearer selection flow from the surveillance pool to the final 12 pairs.

The reviewers asked about how the final 12 were derived from the larger 2024 AEFI reports. The authors say they added a flow diagram, which is good, but this needs to be very explicit and easy to follow in the manuscript.

State very clearly that absence of significant association does not mean absence of risk.

The revised paper is better on this point already, but it should say plainly that the study may have missed true associations because of low power, misclassification, and recall bias.

Move zero-variation variables to descriptive text or supplement.

Since cold-chain variables could not be analytically estimated, keeping them minimal will tighten the paper.

[Note: HTML markup is below. Please do not edit.]

Reviewers' comments:

Reviewer’s Responses to Questions

**Comments to the Author**

1. If the authors have adequately addressed your comments raised in a previous round of review and you feel that this manuscript is now acceptable for publication, you may indicate that here to bypass the “Comments to the Author” section, enter your conflict of interest statement in the “Confidential to Editor” section, and submit your "Accept" recommendation.

Reviewer #3: All comments have been addressed

**********

2. Is the manuscript technically sound, and do the data support the conclusions?

The manuscript must describe a technically sound piece of scientific research with data that supports the conclusions. Experiments must have been conducted rigorously, with appropriate controls, replication, and sample sizes. The conclusions must be drawn appropriately based on the data presented.

Reviewer #3: Partly

**********

3. Has the statistical analysis been performed appropriately and rigorously?

Reviewer #3: N/A

**********

4. Have the authors made all data underlying the findings in their manuscript fully available?

The PLOS Data policy requires authors to make all data underlying the findings described in their manuscript fully available without restriction, with rare exception (please refer to the Data Availability Statement in the manuscript PDF file). The data should be provided as part of the manuscript or its supporting information, or deposited to a public repository. For example, in addition to summary statistics, the data points behind means, medians and variance measures should be available. If there are restrictions on publicly sharing data—e.g. participant privacy or use of data from a third party—those must be specified.

Reviewer #3: Yes

**********

5. Is the manuscript presented in an intelligible fashion and written in standard English?

PLOS ONE does not copyedit accepted manuscripts, so the language in submitted articles must be clear, correct, and unambiguous. Any typographical or grammatical errors should be corrected at revision, so please note any specific errors here.

Reviewer #3: Yes

**********

6. Review Comments to the Author

Please use the space provided to explain your answers to the questions above. You may also include additional comments for the author, including concerns about dual publication, research ethics, or publication ethics. (Please upload your review as an attachment if it exceeds 20,000 characters)

Reviewer #3: Thank you for the careful and thorough revisions. You have effectively addressed the major methodological and statistical concerns, and the manuscript is now clearer, more transparent, and methodologically sound. That said, a few minor issues related to formatting, presentation, and references still need attention before the manuscript can move to final publication.

a. Table and Figure Captions

In your response to the reviewers, you mentioned that the table titles were simplified. However, the revised manuscript still includes repetitive and informal wording in several captions. For example, captions such as “Table 1. This is the Table 1 Operational definitions of exposure variables” appear throughout the manuscript, with similar phrasing used for other tables and figures (e.g., “This is Table 2…”, “This is Fig 1…”).

Therefore, please check and remove phrases such as “This is the Table X” or “This is Fig X” from all captions to conform to standard academic formatting.

b. Bullet points

The current manuscript rely too heavily on bullet points, which makes the content fragmented and less fluid. The author is encouraged to consider rewriting these points as complete sentences or integrating them into coherent paragraphs. This approach would improve the flow, enhance academic tone, and make the feedback easier for readers to follow.

c. Reference List Errors

The reference list contains several errors that appear to result from automated citation extraction and require manual correction. As an illustration, the World Health Organization appears with an incorrect abbreviation, "World Health, O.," in citations 8, 9, 17, 19, and 24. Furthermore, the author’s name in reference 3 is misspelled as "S.C.J.C.I.D. Hadler". Also, there’s no author or issuing organization specified for Reference 10.

Authors must undertake a careful review and correction of the reference list to guarantee that all listed authors, organizations, and URLs are precise and comprehensive.

d. Presentation of the Multivariable Model (minor recommendation)

Although you appropriately acknowledge the limitations of the multivariable conditional logistic regression presented in Table 6, including the small sample of 12 matched pairs, running three independent variables in the same model still presents a substantial risk of overfitting.

For enhanced clarity and a sharper methodological focus in the manuscript, you should think about moving Table 6 and its relevant text to the Supplementary Material. This would allow the main manuscript to emphasize the more appropriate exact McNemar test results (Table 5) and reduce the possibility that readers over-interpret the unstable multivariable estimates.

**********

7. PLOS authors have the option to publish the peer review history of their article (what does this mean?). If published, this will include your full peer review and any attached files.

If you choose “no”, your identity will remain anonymous but your review may still be made public.

**Do you want your identity to be public for this peer review?**   For information about this choice, including consent withdrawal, please see our Privacy Policy.

Reviewer #3: No

**********

[NOTE: If reviewer comments were submitted as an attachment file, they will be attached to this email and accessible via the submission site. Please log into your account, locate the manuscript record, and check for the action link "View Attachments". If this link does not appear, there are no attachment files.]

To ensure your figures meet our technical requirements, please review our figure guidelines: https://journals.plos.org/plosone/s/figures

You may also use PLOS’s free figure tool, NAAS, to help you prepare publication quality figures: https://journals.plos.org/plosone/s/figures#loc-tools-for-figure-preparation

NAAS will assess whether your figures meet our technical requirements by comparing each figure against our figure specifications.

# Author comment: Author response to Decision Letter 2

**DOI:** 10.1371/journal.pone.0351879.r004

30 Mar 2026

Response to Reviewers' Comments

Manuscript Title: Case-control study of risk factors for abscess development following hepatitis B vaccination in children in Timor-Leste, 2024

Dear Editor and Reviewers,

Thank you for the opportunity to revise our manuscript and for the thoughtful and constructive feedback provided during the review process. We have carefully considered all comments and have revised the manuscript accordingly. Below, we provide a point-by-point response to each comment. All changes have been made using track changes in the revised manuscript, and page and line numbers refer to the clean version of the revised document.

We believe these revisions have substantially strengthened the manuscript and hope it is now suitable for publication in PLOS ONE.

Response to Editor and Reviewers

Reviewer Comment 1: Recheck the antiseptic analysis from the raw matched-pair counts. This is the biggest issue. The authors should present the discordant pair counts explicitly and make sure the matched odds ratio, confidence interval, and McNemar p-value all come from the same data and method. If the mOR is truly 1.00, the p-value likely needs correction.

Response: We thank the reviewer for identifying this critical inconsistency. Upon re-examination, we confirmed that the discordant pairs for antiseptic application were indeed 1 and 1, which yields a matched odds ratio of 1.00 and an exact McNemar p-value of 1.000. The previously reported p-value of 0.006 was a transcription error. We have corrected this in Table 4 (page 17, line 278) and have verified that all other p-values in the table are accurate. We appreciate the reviewer’s diligence in catching this error.

Reviewer Comment 2: Consider removing the conditional logistic regression entirely. With only 12 matched pairs, even a 3-variable model is still fragile. The paper would probably be stronger if it relied mainly on descriptive matched analysis and stated clearly that the study was underpowered for robust multivariable inference.

Response: We agree with the reviewer’s assessment. We have removed the conditional logistic regression analysis entirely from the manuscript. The results now focus exclusively on the exact McNemar test for matched bivariate associations, which is more appropriate given the small sample size. We have also strengthened the language throughout the Discussion and Limitations sections to emphasize that the study was underpowered for robust multivariable inference and that findings should be interpreted as exploratory and hypothesis-generating. These revisions can be found in the Statistical Analysis section (page 13, lines 224), the Discussion (page 19, lines 302 and the Strengths and Limitations section (page 21, lines 350).

Reviewer Comment 3: Reframe the study aim slightly. Instead of "identify risk factors," use language like "explore potential factors associated with abscess occurrence during an AEFI investigation." That wording better matches what the data can support.

Response: We have revised the study aim throughout the manuscript to reflect the exploratory nature of this investigation. Specifically:

In the Abstract (page 2, lines 22–24), we now state: "This study aimed to explore potential factors associated with abscess formation to inform improvements in immunization practices."

In the Introduction (page 7, lines 130–132), we have revised the final sentence to read: "This study aimed to explore potential factors associated with abscess occurrence during an AEFI investigation following hepatitis B vaccination using multidose vials in children in Timor-Leste."

Reviewer Comment 4: Add a clearer selection flow from the surveillance pool to the final 12 pairs. The reviewers asked about how the final 12 were derived from the larger 2024 AEFI reports. The authors say they added a flow diagram, which is good, but this needs to be very explicit and easy to follow in the manuscript.

Response: We have added Figure 2 (page 10, lines 184–186), which provides a clear visual representation of the participant selection process. The flow diagram shows the stepwise derivation of the 12 matched pairs from the initial 62 AEFI reports, including the application of eligibility criteria and matching requirements. We have also expanded the accompanying text in the Study Cases section (page 9, lines 161–200) to describe the selection process in detail. We believe this fully addresses the reviewer’s concern regarding transparency of the selection process.

Reviewer Comment 5: State very clearly that absence of significant association does not mean absence of risk. The revised paper is better on this point already, but it should say plainly that the study may have missed true associations because of low power, misclassification, and recall bias.

Response: We have further strengthened this important point. In the Discussion (page 19, lines 312–319), we have added a dedicated paragraph that explicitly states:

"Importantly, the absence of statistically significant associations in this study should not be interpreted as evidence of absence of risk. Given the very small number of matched pairs, this study had limited statistical power to detect anything other than very large effects. As a result, true associations between vaccination practices or recipient characteristics and abscess formation may have been missed. In addition, potential outcome misclassification and recall bias in the retrospective assessment of exposures could have further attenuated observed associations toward the null. The findings should therefore be interpreted as exploratory and hypothesis‑generating rather than definitive."

Reviewer Comment 6: Move zero-variation variables to descriptive text or supplement. Since cold-chain variables could not be analytically estimated, keeping them minimal will tighten the paper.

Response: We agree with the reviewer’s suggestion to streamline the manuscript. We have moved the cold-chain variables (temperature monitoring and vaccine storage conditions) out of the analytical table and now describe them only in the text. Specifically, we state in the Results section (page 16, lines 269–374):

"Vaccine storage and handling practices were uniformly documented as compliant across all participating facilities. For both cases and controls, temperature monitoring logs indicated regular documentation, and physical storage conditions met national cold chain requirements. As no variability was observed in these indicators, they were excluded from the matched analysis."

Additional Revisions

In addition to the changes requested by the reviewer, we have made the following minor revisions to improve manuscript clarity:

Table 4: We verified all discordant pair counts and p-values to ensure consistency with the exact McNemar test calculations. All values are now accurate and internally consistent (page 17, lines 278).

Strengths and Limitations: We added a brief statement acknowledging the potential for selection bias due to the matching requirements (page 21, lines 351–378). While not explicitly requested, we believe this addition further enhances transparency regarding the study’s limitations.

Reviewer Comment Action Taken Location (Page, Lines)

Correct antiseptic p-value Corrected from 0.006 to 1.000 in Table 4 Page 17, line 278

Remove conditional logistic regression Removed entirely; analysis now focuses on exact McNemar test -

Reframe study aim Revised aim to exploratory language in Abstract and Introduction Page 2, lines 22–24; Page 7, lines 130–132

Add clearer selection flow Added Figure 2 and expanded text Page 9, lines 161-200

Clarify absence of significance ≠ absence of risk Added dedicated paragraph in Discussion Page 19, lines 312–319

Move zero-variation variables Described cold-chain variables in text only; excluded from analysis Page 16, lines 270–274

Selection bias acknowledgment (additional) Added statement in Strengths and Limitations Page 21, lines 350–378

Conclusion

We are grateful for the reviewers' insightful comments, which have helped us substantially improve the clarity, rigor, and transparency of our manuscript. We believe the revised version now meets the high standards of PLOS ONE and respectfully submit it for your final consideration.

Corresponding author:

Mariano da Silva Marques

Instituto Nacional da Saúde Pública Timor-Leste

Email: dr.marquess31@gmail.com

Attachment Submitted filename: Response to Reviewers.pdf

# Peer review: Decision Letter 2

**DOI:** 10.1371/journal.pone.0351879.r005

**Authors:** Morufu Olalekan Raimi

11 May 2026

PONE-D-26-02442R2

Case-control study of risk factors for abscess development following hepatitis B vaccination in children in Timor-Leste, 2024

PLOS One

Dear Dr. da Silva Marques,

Thank you for submitting your manuscript to PLOS ONE. After careful consideration, we feel that it has merit but does not fully meet PLOS ONE’s publication criteria as it currently stands. Therefore, we invite you to submit a revised version of the manuscript that addresses the points raised during the review process.

Please submit your revised manuscript by Jun 25 2026 11:59PM. If you will need more time than this to complete your revisions, please reply to this message or contact the journal office at plosone@plos.org. When you're ready to submit your revision, log on to https://www.editorialmanager.com/pone/ and select the ’submissions Needing Revision' folder to locate your manuscript file.

Please include the following items when submitting your revised manuscript:

- A letter that responds to each point raised by the academic editor and reviewer(s). You should upload this letter as a separate file labeled 'Response to Reviewers'.
- A marked-up copy of your manuscript that highlights changes made to the original version. You should upload this as a separate file labeled 'Revised Manuscript with Track Changes'.
- An unmarked version of your revised paper without tracked changes. You should upload this as a separate file labeled 'Manuscript'.

If you would like to make changes to your financial disclosure, please include your updated statement in your cover letter. Guidelines for resubmitting your figure files are available below the reviewer comments at the end of this letter.

If applicable, we recommend that you deposit your laboratory protocols in protocols.io to enhance the reproducibility of your results. Protocols.io assigns your protocol its own identifier (DOI) so that it can be cited independently in the future. For instructions see: https://journals.plos.org/plosone/s/submission-guidelines#loc-laboratory-protocols. Additionally, PLOS ONE offers an option for publishing peer-reviewed Lab Protocol articles, which describe protocols hosted on protocols.io. Read more information on sharing protocols at https://plos.org/protocols?utm_medium=editorial-email&utm_source=authorletters&utm_campaign=protocols.

As the corresponding author, your ORCID iD is verified in the submission system and will appear in the published article. PLOS supports the use of ORCID, and we encourage all coauthors to register for an ORCID iD and use it as well. Please encourage your coauthors to verify their ORCID iD within the submission system before final acceptance, as unverified ORCID iDs will not appear in the published article. *Only*   the individual author can complete the verification step; PLOS staff *cannot*   verify ORCID iDs on behalf of authors.

We look forward to receiving your revised manuscript.

Kind regards,

Morufu Olalekan Raimi, Ph.D

Academic Editor

PLOS One

Journal Requirements:

If the reviewer comments include a recommendation to cite specific previously published works, please review and evaluate these publications to determine whether they are relevant and should be cited. There is no requirement to cite these works unless the editor has indicated otherwise.

**Additional Editor Comments:**

PLOS ONE Editorial Decision

Manuscript ID: PONE-D-26-02442R2

Title: Case-control study of risk factors for abscess development following hepatitis B vaccination in children in Timor-Leste, 2024

Corresponding Author: Dr. Mariano da Silva Marques

Decision: Major Revision Required (not yet acceptable for publication)

OVERALL ASSESSMENT

This manuscript addresses a programmatically critical question, risk factors for injection-site abscesses after hepatitis B birth-dose vaccination in a low-resource setting. The authors have made considerable effort to address reviewer concerns from the previous round, particularly by correcting statistical errors, reframing the study as exploratory, and adding transparency around case selection. However, despite these improvements, several fundamental problems remain that preclude acceptance in the current form. The central issue is not the small sample size per se, rare event investigations are inherently small, but rather a persistent mismatch between what the data can support and what the manuscript still implies, combined with unresolved inconsistencies in the presentation of the primary analysis.

UNRESOLVED CRITICAL ISSUES

1. Statistical analysis remains misaligned with the study design and sample size

The authors removed conditional logistic regression but retained the exact McNemar test. However, Table 4 (page 27) still reports p-values, mORs, and CIs as if these estimates are meaningful. With only 12 matched pairs and discordant counts of 1, 2, or 4, these p-values and CIs are fundamentally uninformative, not just “wide.” The continued presentation of p=1.000 for multiple variables (antiseptic, vaccinator training, experience) creates a misleading impression of no effect when in fact the data contain no information about these associations.

Required action: Either remove all inferential statistics (p-values, CIs) from Table 4 entirely and present only discordant pair counts, or add a bolded, explicit caveat directly beneath the table stating: “Due to the very small number of discordant pairs, these confidence intervals and p-values are statistically unstable and should not be interpreted as evidence for or against association.”

2. The antiseptic use error has been corrected procedurally but not interpretively

The corrected mOR=1.00 and p=1.000 are now correct. However, the authors retain text in the Discussion (page 29, lines 324-328 and elsewhere) discussing “non-significant trends” and “point estimates reinforcing potential importance” for variables that have virtually no discordant pairs. For a variable with 1:1 discordant pairs, an mOR of 1.00 does not “reinforce” anything. This over-interpretation must be removed.

3. The abstract still overstates the contribution

Line 39-44 (page 13) states: “In this exploratory matched case-control study, no statistically significant risk factors…were identified. The findings highlight the statistical uncertainty inherent in analyzing rare events with small samples.” This is acceptable. But the preceding lines (12-14) in the Results section of the abstract still present mORs with CIs as if they are informative. The abstract should lead with the limitation, not bury it.

4. Persistent internal inconsistency between text and tables

• Page 16, lines 270–274 state cold chain variables were excluded from analysis. Good. But Table 4 (page 27) does not indicate this exclusion clearly; it simply omits them. A footnote is needed.

• The hypothesised causal pathway (Figure 1) remains very general and does not map directly onto the variables actually measured. Consider moving to supplementary material or condensing.

________________________________________

Required Revisions (Moderate, but Non-Negotiable)

Section Required Action

Abstract Remove presentation of individual mORs and CIs from Results. Replace with: “None of the examined variables showed statistically significant associations in matched bivariate analysis, and confidence intervals were extremely wide, reflecting the small sample size (12 pairs).”

Table 4 Add a prominent footnote: “Due to the very small number of discordant pairs (≤4 for all variables), matched odds ratios and confidence intervals are statistically unstable. These estimates should not be interpreted as measures of effect magnitude.”

Discussion (page 29–30) Remove speculative language about “trends” for previously opened vials (mOR=4.00 with CI crossing null). Replace with: “The discordant pair pattern for multidose vial status (4 vs 1) is compatible with a wide range of possible effect sizes, from protective to strongly harmful.”

Strengths and Limitations (page 31–32) Add one sentence: “Because all inferential estimates are highly unstable, this study cannot reliably confirm or refute any specific risk factor. Its primary utility is hypothesis generation and reinforcement of safe practices.”

Conclusion (page 32–33) Remove the sentence beginning “While vaccination from a previously opened multidose vial showed a nonsignificant trend…” This is overinterpretation. Conclude instead on programmatic recommendations only.

What the Study Actually Contributes (and Does Not)

Does contribute: A transparent, honest documentation of an AEFI investigation in a low-resource setting where 12 matched pairs were analyzable, with no clear signal for any single factor. The flow diagram, case definitions, and data collection process are now clearly described. This has local surveillance value.

Does NOT contribute: Any reliable estimate of association, any meaningful p-value, any “trend” that can be interpreted, or any evidence to guide practice change beyond standard safe injection protocols.

The authors should reframe the final manuscript as a case-series with matched comparators for descriptive purposes, not as an analytic case-control study. The current framing still implies more analytic weight than the data can bear.

RECOMMENDATION

Major revision required (not moderate, because the interpretive framing remains problematic despite prior revisions). The manuscript will be suitable for acceptance only if:

1. All inferential overstatement is removed from abstract, discussion, and conclusion.

2. Table 4 is explicitly caveated for instability.

3. The study is reframed as descriptive/exploratory without implication of effect estimation.

If the authors can make these adjustments within 4 weeks, I will recommend acceptance. Otherwise, the manuscript cannot be published in its current form.

Dr. Morufu Olalekan Raimi Ph.D

PLOS ONE Editorial Board

[Note: HTML markup is below. Please do not edit.]

Reviewers' comments:

Reviewer’s Responses to Questions

**Comments to the Author**

1. If the authors have adequately addressed your comments raised in a previous round of review and you feel that this manuscript is now acceptable for publication, you may indicate that here to bypass the “Comments to the Author” section, enter your conflict of interest statement in the “Confidential to Editor” section, and submit your "Accept" recommendation.

Reviewer #3: (No Response)

**********

2. Is the manuscript technically sound, and do the data support the conclusions?

The manuscript must describe a technically sound piece of scientific research with data that supports the conclusions. Experiments must have been conducted rigorously, with appropriate controls, replication, and sample sizes. The conclusions must be drawn appropriately based on the data presented.

Reviewer #3: No

**********

3. Has the statistical analysis been performed appropriately and rigorously?

Reviewer #3: Yes

**********

4. Have the authors made all data underlying the findings in their manuscript fully available?

The PLOS Data policy requires authors to make all data underlying the findings described in their manuscript fully available without restriction, with rare exception (please refer to the Data Availability Statement in the manuscript PDF file). The data should be provided as part of the manuscript or its supporting information, or deposited to a public repository. For example, in addition to summary statistics, the data points behind means, medians and variance measures should be available. If there are restrictions on publicly sharing data—e.g. participant privacy or use of data from a third party—those must be specified.

Reviewer #3: Yes

**********

5. Is the manuscript presented in an intelligible fashion and written in standard English?

PLOS ONE does not copyedit accepted manuscripts, so the language in submitted articles must be clear, correct, and unambiguous. Any typographical or grammatical errors should be corrected at revision, so please note any specific errors here.

Reviewer #3: Yes

**********

6. Review Comments to the Author

Please use the space provided to explain your answers to the questions above. You may also include additional comments for the author, including concerns about dual publication, research ethics, or publication ethics. (Please upload your review as an attachment if it exceeds 20,000 characters)

Reviewer #3: The authors have transparently addressed the previous statistical concerns by appropriately removing the overfitted multivariable conditional logistic regression model and correcting the exact McNemar test calculations (e.g., adjusting the antiseptic application p-value to 1.000).

However, while the statistical analysis is now honest and appropriately executed, these necessary corrections have brought insurmountable methodological limitations to the forefront. Unfortunately, the underlying study design and data structure limit the manuscript’s ability to meet the standards of a technically robust scientific study.

Below are the detailed major methodological concerns:

1. Severe Overmatching Bias The most critical limitation lies in the matching criteria. By matching cases and controls 1:1 based on the exact same vaccination facility and the same vaccination date (±7 days), the study design inadvertently introduced severe overmatching. This matching strategy essentially forced the case and control to share the exact same cold chain environment (e.g., the same refrigerator), potentially the same multidose vial, and likely the same vaccinator. Consequently, this artificially eliminated the variance in the core exposure variables the study aimed to investigate (which explains why cold chain maintenance showed 100% uniformity across both groups). When exposure variance is designed out of the study, it becomes impossible to detect true risk factors.

2. Severely Underpowered / Null Findings With a final sample size of only 12 matched pairs (N=24), the study lacks the statistical power required to answer its research question. Following the appropriate statistical corrections in this revision, all variables yielded non-significant results (all p-values > 0.05) with extremely wide and unstable confidence intervals. While there is absolute value in publishing null findings, a study must first have the adequate statistical power to detect a meaningful difference if one exists. A null result derived from a highly underpowered sample of 12 pairs limits the ability to draw robust or generalizable conclusions.

3. Susceptibility to Severe Recall Bias The study relies on retrospective data collection, interviewing caregivers and healthcare workers in late 2025 regarding minor procedural details of a vaccination event that occurred in 2024. Asking individuals to accurately recall specific, routine micro-practices from a year prior—such as whether a nurse wiped a specific infant’s leg with an antiseptic swab or whether a multidose vial was newly opened for that exact dose—introduces a profound risk of recall and information bias. As the authors rightly acknowledged in the limitations, this likely led to significant non-differential misclassification.

**********

7. PLOS authors have the option to publish the peer review history of their article (what does this mean?). If published, this will include your full peer review and any attached files.

If you choose “no”, your identity will remain anonymous but your review may still be made public.

**Do you want your identity to be public for this peer review?**   For information about this choice, including consent withdrawal, please see our Privacy Policy.

Reviewer #3: No

**********

[NOTE: If reviewer comments were submitted as an attachment file, they will be attached to this email and accessible via the submission site. Please log into your account, locate the manuscript record, and check for the action link "View Attachments". If this link does not appear, there are no attachment files.]

To ensure your figures meet our technical requirements, please review our figure guidelines: https://journals.plos.org/plosone/s/figures

You may also use PLOS’s free figure tool, NAAS, to help you prepare publication quality figures: https://journals.plos.org/plosone/s/figures#loc-tools-for-figure-preparation.

NAAS will assess whether your figures meet our technical requirements by comparing each figure against our figure specifications.

# Author comment: Author response to Decision Letter 3

**DOI:** 10.1371/journal.pone.0351879.r006

20 May 2026

Response to Reviewers' Comments

Manuscript Title: Case-control study of risk factors for abscess development following hepatitis B vaccination in children in Timor-Leste, 2024

Manuscript ID: PONE-D-26-02442R2

Dear Editor and Reviewers,

Thank you for your detailed and constructive review of our manuscript. We appreciate your recognition that we have made considerable efforts to address previous reviewer concerns, including correcting statistical errors, reframing the study as exploratory, and adding transparency around case selection.

We have carefully considered each of your required revisions. Below we respond point by point and indicate where changes have been made in the revised manuscript. All changes are clearly marked in bold in the revised document.

PART 1: Response to Editorial Office Summary

Editorial Comment (Overall Assessment):

This manuscript addresses a programmatically critical question... However, despite these improvements, several fundamental problems remain that preclude acceptance in the current form. The central issue is not the small sample size per se... but rather a persistent mismatch between what the data can support and what the manuscript still implies, combined with unresolved inconsistencies in the presentation of the primary analysis.

Author Response: We thank the Editor for this honest and constructive assessment. We have now thoroughly revised the manuscript to eliminate the mismatch between the data and the interpretation. All inferential overstatements have been removed, and the manuscript is now explicitly framed as descriptive and hypothesis-generating only. Specific changes are detailed in response to each point below.

PART 2: Response to Reviewer Comments (Dr. Morufu Olalekan Raimi)

UNRESOLVED CRITICAL ISSUES

Comment 1: Statistical analysis remains misaligned with the study design and sample size

Reviewer said:

The authors removed conditional logistic regression but retained the exact McNemar test. However, Table 4 (page 27) still reports p-values, mORs, and CIs as if these estimates are meaningful. With only 12 matched pairs and discordant counts of 1, 2, or 4, these p-values and CIs are fundamentally uninformative, not just "wide." The continued presentation of p=1.000 for multiple variables (antiseptic, vaccinator training, experience) creates a misleading impression of no effect when in fact the data contain no information about these associations.

Required action: Either remove all inferential statistics (p-values, CIs) from Table 4 entirely and present only discordant pair counts, or add a bolded, explicit caveat directly beneath the table stating: "Due to the very small number of discordant pairs, these confidence intervals and p-values are statistically unstable and should not be interpreted as evidence for or against association."

Author Response: Accepted. We have added the requested bolded footnote directly beneath Table 4 exactly as worded.

Action taken:

A bolded footnote has been added: "Due to the very small number of discordant pairs (≤4 for all variables), matched odds ratios and confidence intervals are statistically unstable. These estimates should not be interpreted as measures of effect magnitude."

The existing footnote about cold chain variables has been retained above it.

Location in revised manuscript: Table 4, footnotes

Comment 2: The antiseptic use error has been corrected procedurally but not interpretively

Reviewer said:

The corrected mOR=1.00 and p=1.000 are now correct. However, the authors retain text in the Discussion (page 29, lines 324-328 and elsewhere) discussing "non-significant trends" and "point estimates reinforcing potential importance" for variables that have virtually no discordant pairs. For a variable with 1:1 discordant pairs, an mOR of 1.00 does not "reinforce" anything. This over-interpretation must be removed.

Author Response: Accepted. We have removed all speculative language about "trends" and "point estimates reinforcing potential importance" from the Discussion section.

Action taken:

The sentence containing "nonsignificant trend" has been removed.

The phrase "point estimates reinforcing potential importance" has been removed.

We have reviewed the entire Discussion section to ensure no other over-interpretive language remains.

Location in revised manuscript: Discussion, paragraph 1 (previously lines 324–328)

Comment 3: The abstract still overstates the contribution

Reviewer said:

Line 39-44 (page 13) states: "In this exploratory matched case-control study, no statistically significant risk factors…were identified. The findings highlight the statistical uncertainty inherent in analyzing rare events with small samples." This is acceptable. But the preceding lines (12-14) in the Results section of the abstract still present mORs with CIs as if they are informative. The abstract should lead with the limitation, not bury it.

Required action: Remove presentation of individual mORs and CIs from Results. Replace with: "None of the examined variables showed statistically significant associations in matched bivariate analysis, and confidence intervals were extremely wide, reflecting the small sample size (12 pairs)."

Author Response: Accepted. We have revised the abstract Results section as requested.

Action taken:

Individual mORs and CIs have been removed entirely.

Replaced with the exact wording: "None of the examined variables showed statistically significant associations in matched bivariate analysis, and confidence intervals were extremely wide, reflecting the small sample size (12 pairs)."

Location in revised manuscript: Abstract, Results section

Comment 4: Persistent internal inconsistency between text and tables

Reviewer said:

Page 16, lines 270–274 state cold chain variables were excluded from analysis. Good. But Table 4 (page 27) does not indicate this exclusion clearly; it simply omits them. A footnote is needed.

The hypothesised causal pathway (Figure 1) remains very general and does not map directly onto the variables actually measured. Consider moving to supplementary material or condensing.

Author Response: Accepted on both points.

Action taken (cold chain variables):

A footnote has been added to Table 4 indicating: "aCold chain variables (temperature monitoring and physical storage conditions) were excluded from matched analysis because no discordant pairs were observed across matched case–control pairs."

Action taken (causal pathway figure):

Figure 1 (causal pathway diagram) has been removed entirely from the manuscript, as it does not map directly onto the measured variables.

All references to Figure 1 in the text have been deleted.

The case selection flow diagram (formerly Figure 2) has been retained and renumbered as Figure 1, as it provides transparent documentation of the case selection process.

Location in revised manuscript: Table 4 footnotes; Methods section (Figure 1, renumbered)

REQUIRED REVISIONS (Moderate, but Non-Negotiable)

Required Revision 1: Abstract

Reviewer said:

Remove presentation of individual mORs and CIs from Results. Replace with: "None of the examined variables showed statistically significant associations in matched bivariate analysis, and confidence intervals were extremely wide, reflecting the small sample size (12 pairs)."

Author Response: Completed as detailed above.

Location: Abstract, Results section

Required Revision 2: Table 4

Reviewer said:

add a prominent footnote: "Due to the very small number of discordant pairs (≤4 for all variables), matched odds ratios and confidence intervals are statistically unstable. These estimates should not be interpreted as measures of effect magnitude."

Author Response: Completed as detailed above.

Location: Table 4, bolded footnote beneath table

Required Revision 3: Discussion (page 29–30)

Reviewer said:

Remove speculative language about "trends" for previously opened vials (mOR=4.00 with CI crossing null). Replace with: "The discordant pair pattern for multidose vial status (4 vs 1) is compatible with a wide range of possible effect sizes, from protective to strongly harmful."

Author Response: Completed as detailed above.

Location: Discussion, paragraph 1

Required Revision 4: Strengths and Limitations (page 31–32)

Reviewer said:

Add one sentence: "Because all inferential estimates are highly unstable, this study cannot reliably confirm or refute any specific risk factor. Its primary utility is hypothesis generation and reinforcement of safe practices."

Author Response: Completed.

Action taken: The requested sentence has been added verbatim to the Strengths and Limitations section, immediately following the discussion of small sample size and unstable estimates.

Location: Strengths and Limitations, paragraph 2

Required Revision 5: Conclusion (page 32–33)

Reviewer said:

Remove the sentence beginning "While vaccination from a previously opened multidose vial showed a nonsignificant trend…" This is overinterpretation. Conclude instead on programmatic recommendations only.

Author Response: Completed.

Action taken:

The offending sentence has been removed entirely.

The Conclusion now focuses solely on programmatic recommendations.

Location: Conclusion

PART 3: Summary Table of All Changes

Section Reviewer Request Action Taken

Abstract Results Remove mORs and CIs; replace with requested sentence Completed

Table 4 Add bolded footnote about statistical instability Completed

Table 4 Add footnote about cold chain variable exclusion Completed

Discussion Remove "trends" language; replace with neutral statement Completed

Strengths & Limitations Add sentence about inability to confirm/refute risk factors Completed

Conclusion Remove overinterpretation of multidose vial finding Completed

Figures Remove causal pathway figure (Figure 1) Completed

Figures Renumber case selection flow diagram as Figure 1 Completed

PART 4: What the Study Actually Contributes (Acknowledgment)

We acknowledge and accept the reviewer’s characterization:

Does contribute:

A transparent, honest documentation of an AEFI investigation in a low-resource setting

Clear description of case definitions, flow diagram, and data collection process

Local surveillance value

Does NOT contribute:

Any reliable estimate of association

Any meaningful p-value

Any "trend" that can be interpreted

Any evidence to guide practice change beyond standard safe injection protocols

The manuscript has been reframed accordingly.

PART 5: Conclusion

We believe the revised manuscript now fully addresses all concerns raised by the reviewer and the Editor. All inferential overstatement has been removed, Table 4 is properly caveated, the causal pathway figure has been removed, and the study is explicitly framed as descriptive/hypothesis-generating without implication of effect estimation.

We thank the reviewer and Editor for their rigorous and constructive feedback, which has substantially improved the clarity, honesty, and scientific integrity of our manuscript.

We look forward to your final decision.

Attachments:

Revised manuscript with track changes

Clean revised manuscript

Corresponding author:

Mariano da Silva Marques

Instituto Nacional da Saúde Pública Timor-Leste

Email: dr.marquess31@gmail.com

Attachment Submitted filename: Response_to_Reviewers_auresp_3.pdf

# Editor report: Decision Letter 3

**DOI:** 10.1371/journal.pone.0351879.r007

**Authors:** Morufu Olalekan Raimi

1 Jun 2026

PONE-D-26-02442R3

Case-control study of risk factors for abscess development following hepatitis B vaccination in children in Timor-Leste, 2024

PLOS One

Dear Dr. da Silva Marques,

Thank you for submitting your manuscript to PLOS ONE. After careful consideration, we feel that it has merit but does not fully meet PLOS ONE’s publication criteria as it currently stands. Therefore, we invite you to submit a revised version of the manuscript that addresses the points raised during the review process.

Please submit your revised manuscript by Jul 16 2026 11:59PM. If you will need more time than this to complete your revisions, please reply to this message or contact the journal office at plosone@plos.org. When you're ready to submit your revision, log on to https://www.editorialmanager.com/pone/ and select the ’submissions Needing Revision' folder to locate your manuscript file.

Please include the following items when submitting your revised manuscript:

- A letter that responds to each point raised by the academic editor and reviewer(s). You should upload this letter as a separate file labeled 'Response to Reviewers'.
- A marked-up copy of your manuscript that highlights changes made to the original version. You should upload this as a separate file labeled 'Revised Manuscript with Track Changes'.
- An unmarked version of your revised paper without tracked changes. You should upload this as a separate file labeled 'Manuscript'.

If you would like to make changes to your financial disclosure, please include your updated statement in your cover letter. Guidelines for resubmitting your figure files are available below the reviewer comments at the end of this letter.

If applicable, we recommend that you deposit your laboratory protocols in protocols.io to enhance the reproducibility of your results. Protocols.io assigns your protocol its own identifier (DOI) so that it can be cited independently in the future. For instructions see: https://journals.plos.org/plosone/s/submission-guidelines#loc-laboratory-protocols. Additionally, PLOS ONE offers an option for publishing peer-reviewed Lab Protocol articles, which describe protocols hosted on protocols.io. Read more information on sharing protocols at https://plos.org/protocols?utm_medium=editorial-email&utm_source=authorletters&utm_campaign=protocols.

As the corresponding author, your ORCID iD is verified in the submission system and will appear in the published article. PLOS supports the use of ORCID, and we encourage all coauthors to register for an ORCID iD and use it as well. Please encourage your coauthors to verify their ORCID iD within the submission system before final acceptance, as unverified ORCID iDs will not appear in the published article. *Only*   the individual author can complete the verification step; PLOS staff *cannot*   verify ORCID iDs on behalf of authors.

We look forward to receiving your revised manuscript.

Kind regards,

Morufu Olalekan Raimi, Ph.D

Academic Editor

PLOS One

Journal Requirements:

1. If the reviewer comments include a recommendation to cite specific previously published works, please review and evaluate these publications to determine whether they are relevant and should be cited. There is no requirement to cite these works unless the editor has indicated otherwise.

2. Please review your reference list to ensure that it is complete and correct. If you have cited papers that have been retracted, please include the rationale for doing so in the manuscript text, or remove these references and replace them with relevant current references. Any changes to the reference list should be mentioned in the rebuttal letter that accompanies your revised manuscript. If you need to cite a retracted article, indicate the article’s retracted status in the References list and also include a citation and full reference for the retraction notice.

**Additional Editor Comments:**

PLOS ONE Editorial Decision

Manuscript ID: PONE-D-26-02442R3

Title: Case-control study of risk factors for abscess development following hepatitis B vaccination in children in Timor-Leste, 2024

Corresponding Author: Dr. Mariano da Silva Marques

Decision: Minor Revision (conditional acceptance)

OVERALL ASSESSMENT

The authors have responded substantively and in good faith to the required revisions from the previous round. I acknowledge that they have:

• Removed all inferential overstatements from the abstract, discussion, and conclusion.

• Added the bolded statistical caveat to Table 4, explicitly warning against interpreting p-values, CIs, or mORs as reliable measures.

• Removed the speculative language regarding “trends” and “point estimates reinforcing potential importance.”

• Removed the non-informative causal pathway figure.

• Explicitly reframed the study as descriptive and hypothesis generating.

These changes have substantially improved the manuscript’s transparency and scientific honesty. The remaining issues are minor and do not require re-review.

UNRESOLVED ISSUES (MINOR, REQUIRING FINAL CORRECTIONS)

1. Inconsistent footnote duplication in Table 4

In the revised manuscript (pages 27-28 of the PDF, and again on pages 57-58), Table 4 contains two identical footnotes labeled with the same asterisk symbol. One states:

“Due to the very small number of discordant pairs (≤4 for all variables), matched odds ratios, confidence intervals, and p values are statistically unstable and should not be interpreted as reliable measures of effect magnitude or evidence for or against association.”

The other, also marked with an asterisk, repeats substantially the same content. This duplication is redundant and should be consolidated into a single footnote placed directly beneath the table.

Required action: Keep only one version of the bolded instability footnote. Remove the duplicate. Retain the separate footnote about cold chain variables (marked with a different symbol, e.g., † or a).

2. Minor inconsistency in the Discussion

Page 29 (and page 59) states:

“The discordant pair pattern for multidose vial status (4 vs. 1) is compatible with a very wide range of possible effects, from no association to a substantial increase in risk.”

This is acceptable and appropriately neutral. However, the very next sentence says:

“No meaningful inference can be drawn from this pattern alone.”

The word “alone” is unnecessary because the preceding sentence already establishes that no precise inference is possible. Delete “alone” to avoid any residual suggestion that additional information might salvage the inference.

3. Abstract – redundant phrasing

The abstract’s Results section (page 13, line 39-44) reads:

“None of the examined variables showed statistically significant associations… and confidence intervals were extremely wide, reflecting the small sample size (12 pairs).”

The subsequent sentence in the abstract says:

“The very small number of discordant pairs (≤4 for all comparisons) resulted in substantial statistical instability, precluding any reliable estimation of associations.”

This is repetitive. Combine or remove the second sentence to avoid verbatim overlap. I suggest keeping only:

“None of the examined variables showed statistically significant associations; confidence intervals were extremely wide, reflecting the small sample size (12 pairs) and the very small number of discordant pairs (≤4 for all comparisons).”

WHAT THIS MANUSCRIPT NOW CORRECTLY CONTRIBUTES

• Transparent documentation of a real world AEFI investigation in a low resource setting.

• A clear case definition, flow diagram, and matching protocol.

• An explicit acknowledgment that no reliable effect estimates can be derived.

• Local surveillance value for Timor Leste’s immunization program.

What it does not claim (and no longer implies):

Any reliable odds ratio, meaningful p value, interpretable trend, or evidence to change practice beyond standard safe injection protocols.

RECOMMENDATION

Minor Revision. The manuscript is not acceptable in its current form only due to the three minor issues listed above (duplicate footnote, unnecessary word “alone,” and repetitive abstract phrasing). These can be corrected in 7 days without further external review.

Once the authors:

1. Consolidate the duplicate footnote in Table 4 into a single statement,

2. Delete the word “alone” from the Discussion sentence, and

3. Streamline the abstract’s Results to avoid repetition,

the manuscript will meet PLOS ONE’s publication criteria. I will recommend acceptance immediately upon receipt of the corrected version.

No further statistical re analysis, re framing, or major rewriting is required.

FINAL REMARK TO THE AUTHORS

You have done a commendable job transforming an over interpreted small study into an honest, exploratory, and methodologically transparent report. That shift is rare and valuable. Correct the three minor items above, and this paper will serve as a useful example for other investigators facing similar rare event investigations in low resource settings.

Decision: Minor Revision (7 days)

[Note: HTML markup is below. Please do not edit.]

[NOTE: If reviewer comments were submitted as an attachment file, they will be attached to this email and accessible via the submission site. Please log into your account, locate the manuscript record, and check for the action link "View Attachments". If this link does not appear, there are no attachment files.]

To ensure your figures meet our technical requirements, please review our figure guidelines: https://journals.plos.org/plosone/s/figures

You may also use PLOS’s free figure tool, NAAS, to help you prepare publication quality figures: https://journals.plos.org/plosone/s/figures#loc-tools-for-figure-preparation.

NAAS will assess whether your figures meet our technical requirements by comparing each figure against our figure specifications.

# Author comment: Author response to Decision Letter 4

**DOI:** 10.1371/journal.pone.0351879.r008

1 Jun 2026

Response to Reviewers' Comments

Manuscript Title: Case-control study of risk factors for abscess development following hepatitis B vaccination in children in Timor-Leste, 2024

Manuscript ID: PONE-D-26-02442R3

Dear Editor and Reviewers,

Thank you for your thoughtful and constructive review of our manuscript. We appreciate your recognition of the substantive improvements made in response to previous revisions and your clear guidance on the remaining minor issues. We have addressed each of the three items as requested.

Below, we respond point-by-point to your comments.

Comment 1: Inconsistent footnote duplication in Table 4

Editor’s comment:

"In the revised manuscript (pages 27-28 of the PDF, and again on pages 57-58), Table 4 contains two identical footnotes labeled with the same asterisk symbol... This duplication is redundant and should be consolidated into a single footnote placed directly beneath the table.

Required action: Keep only one version of the bolded instability footnote. Remove the duplicate. Retain the separate footnote about cold chain variables (marked with a different symbol, e.g., † or a)."

Authors' response:

We have consolidated the duplicate footnotes into a single bolded instability statement placed directly beneath Table 4. The cold chain variables footnote now uses a distinct symbol (†) to avoid confusion. The revised Table 4 footnote now reads:

*Due to the very small number of discordant pairs (≤4 for all variables), matched odds ratios, confidence intervals, and p-values are statistically unstable and should not be interpreted as reliable measures of effect magnitude or evidence for or against association.

†Cold chain variables (temperature monitoring and physical storage conditions) were excluded from matched analysis because no discordant pairs were observed across matched case–control pairs.

Action taken: Completed. The duplicate footnote has been removed.

Comment 2: Minor inconsistency in the Discussion

Editor’s comment:

"Page 29 (and page 59) states: 'The discordant pair pattern for multidose vial status (4 vs. 1) is compatible with a very wide range of possible effects, from no association to a substantial increase in risk.' This is acceptable and appropriately neutral. However, the very next sentence says: 'No meaningful inference can be drawn from this pattern alone.' The word 'alone' is unnecessary because the preceding sentence already establishes that no precise inference is possible. Delete 'alone' to avoid any residual suggestion that additional information might salvage the inference."

Authors' response:

We have deleted the word "alone" from the sentence as requested. The revised sentence now reads:

"No meaningful inference can be drawn from this pattern."

Action taken: Completed. The word "alone" has been removed from the Discussion section.

Comment 3: Abstract – redundant phrasing

Editor’s comment:

"The abstract’s Results section (page 13, line 39-44) reads: 'None of the examined variables showed statistically significant associations… and confidence intervals were extremely wide, reflecting the small sample size (12 pairs).' The subsequent sentence in the abstract says: 'The very small number of discordant pairs (≤4 for all comparisons) resulted in substantial statistical instability, precluding any reliable estimation of associations.' This is repetitive. Combine or remove the second sentence to avoid verbatim overlap. I suggest keeping only: 'None of the examined variables showed statistically significant associations; confidence intervals were extremely wide, reflecting the small sample size (12 pairs) and the very small number of discordant pairs (≤4 for all comparisons).'"

Authors' response:

We have streamlined the abstract’s Results section into a single sentence as suggested. The revised text now reads:

"None of the examined variables showed statistically significant associations with injection-site abscess formation; confidence intervals were extremely wide, reflecting the small sample size (12 pairs) and the very small number of discordant pairs (≤4 for all comparisons). Findings should therefore be interpreted as descriptive and hypothesis-generating only.."

Action taken: Completed. The repetitive phrasing has been removed.

Summary of Changes

Editor Comment Action Taken

Duplicate footnote in Table 4 Consolidated into a single bolded instability footnote; retained a separate footnote for cold chain variables using † symbol

Unnecessary word "alone" in Discussion Removed "alone" from the sentence

Repetitive phrasing in Abstract Results Streamlined into a single combined sentence

Declaration

We confirm that:

1. No other changes have been made to the manuscript beyond the three specific corrections requested above.

2. The scientific content, statistical interpretations, and overall framing remain exactly as approved in principle by the Editor.

3. The manuscript is not under consideration elsewhere and has been approved by all authors.

We believe the manuscript now meets PLOS ONE’s publication criteria and respectfully submit this corrected version for final acceptance.

Thank you again for your fair and constructive review, and for your recognition of our efforts to present this small study with appropriate transparency and scientific honesty.

Attachments:

Revised manuscript with track changes

Clean revised manuscript

Corresponding author:

Mariano da Silva Marques

Instituto Nacional da Saúde Pública Timor-Leste

Email: dr.marquess31@gmail.com

Attachment Submitted filename: Response_to_Reviewers_auresp_4.pdf

# Editor report: Decision Letter 4

**DOI:** 10.1371/journal.pone.0351879.r009

**Authors:** Morufu Olalekan Raimi

2 Jun 2026

Case-control study of risk factors for abscess development following hepatitis B vaccination in children in Timor-Leste, 2024

PONE-D-26-02442R4

Dear Authors,

We’re pleased to inform you that your manuscript has been judged scientifically suitable for publication and will be formally accepted for publication once it meets all outstanding technical requirements.

Within one week, you’ll receive an e-mail detailing the required amendments. When these have been addressed, you’ll receive a formal acceptance letter and your manuscript will be scheduled for publication.

An invoice will be generated when your article is formally accepted. Please note, if your institution has a publishing partnership with PLOS and your article meets the relevant criteria, all or part of your publication costs will be covered. Please make sure your user information is up-to-date by logging into Editorial Manager at Editorial Manager® and clicking the ‘Update My Information' link at the top of the page. For questions related to billing, please contact billing support.

If your institution or institutions have a press office, please notify them about your upcoming paper to help maximize its impact. If they’ll be preparing press materials, please inform our press team as soon as possible -- no later than 48 hours after receiving the formal acceptance. Your manuscript will remain under strict press embargo until 2 pm Eastern Time on the date of publication. For more information, please contact onepress@plos.org.

Kind regards,

Morufu Olalekan Raimi, Ph.D

Academic Editor

PLOS One

Additional Editor Comments (optional):

PLOS ONE Editorial Office

Decision Letter – Final Review

Manuscript ID: PONE-D-26-02442R4

Title: Case-control study of risk factors for abscess development following hepatitis B vaccination in children in Timor-Leste, 2024.

Corresponding Author: Dr. Mariano da Silva Marques

Academic Editor: Dr. Morufu Olalekan Raimi

Decision: ACCEPT

Editorial Assessment

The authors have meticulously addressed all three minor issues raised in the previous revision (R3) exactly as requested:

1. Table 4 footnotes – Duplicate instability footnote consolidated into a single bolded statement; cold chain variables footnote now correctly marked with a dagger (†).

2. Discussion – The word “alone” has been removed from the sentence concerning multidose vial discordant pairs.

3. Abstract Results – Redundant phrasing streamlined into a single, clear sentence without loss of meaning.

No other changes have been made to the scientific content, statistical interpretations, or overall framing. The manuscript remains transparent, appropriately cautious, and explicitly hypothesis generating, exactly as approved in principle after the previous round.

Final Verdict

The manuscript now meets all PLOS ONE publication criteria. It provides a methodologically honest account of a real world AEFI investigation in a low resource setting, with clear limitations stated and no overstatements. The work will serve as a useful model for similar rare event studies.

I recommend acceptance without further revision.

Dr. Morufu Olalekan Raimi

Academic Editor, PLOS ONE

Reviewers' comments:

# Editor report: Acceptance letter

**DOI:** 10.1371/journal.pone.0351879.r010

**Authors:** Morufu Olalekan Raimi

PONE-D-26-02442R4

PLOS One

Dear Dr. da Silva Marques,

I'm pleased to inform you that your manuscript has been deemed suitable for publication in PLOS One. Congratulations! Your manuscript is now being handed over to our production team.

At this stage, our production department will prepare your paper for publication. This includes ensuring the following:

* All references, tables, and figures are properly cited

* All relevant supporting information is included in the manuscript submission,

* There are no issues that prevent the paper from being properly typeset

You will receive further instructions from the production team, including instructions on how to review your proof when it is ready. Please keep in mind that we are working through a large volume of accepted articles, so please give us a few days to review your paper and let you know the next and final steps.

Lastly, if your institution or institutions have a press office, please let them know about your upcoming paper now to help maximize its impact. If they'll be preparing press materials, please inform our press team within the next 48 hours. Your manuscript will remain under strict press embargo until 2 pm Eastern Time on the date of publication. For more information, please contact onepress@plos.org.

You will receive an invoice from PLOS for your publication fee after your manuscript has reached the completed accept phase. If you receive an email requesting payment before acceptance or for any other service, this may be a phishing scheme. Learn how to identify phishing emails and protect your accounts at https://explore.plos.org/phishing.

If we can help with anything else, please email us at customercare@plos.org.

Thank you for submitting your work to PLOS ONE and supporting open access.

Kind regards,

PLOS ONE Editorial Office Staff

on behalf of

Prof Morufu Olalekan Raimi

Academic Editor

PLOS One
